# Three-dimensional morphometric analysis reveals time-dependent structural changes in microglia and astrocytes in the central amygdala and hypothalamic paraventricular nucleus of heart failure rats

**DOI:** 10.1186/s12974-020-01892-4

**Published:** 2020-07-23

**Authors:** Ferdinand Althammer, Hildebrando Candido Ferreira-Neto, Myurajan Rubaharan, Ranjan K. Roy, Atit A. Patel, Daniel N. Cox, Javier E. Stern

**Affiliations:** 1 Center for Neuroinflammation and Cardiometabolic Diseases, Georgia State University, Atlanta, USA; 2 Neuroscience Institute, Georgia State University, Atlanta, USA

**Keywords:** Hypothalamus, Amygdala, Microglia, Astrocytes, A1, Behavior, Cytokines, Heart failure, Neuroinflammation, Autonomic

## Abstract

**Background:**

Cardiovascular diseases, including heart failure, are the most common cause of death globally. Recent studies support a high degree of comorbidity between heart failure and cognitive and mood disorders resulting in memory loss, depression, and anxiety. While neuroinflammation in the hypothalamic paraventricular nucleus contributes to autonomic and cardiovascular dysregulation in heart failure, mechanisms underlying cognitive and mood disorders in this disease remain elusive. The goal of this study was to quantitatively assess markers of neuroinflammation (glial morphology, cytokines, and A1 astrocyte markers) in the central amygdala, a critical forebrain region involved in emotion and cognition, and to determine its time course and correlation to disease severity during the progression of heart failure.

**Methods:**

We developed and implemented a comprehensive microglial/astrocyte profiler for precise three-dimensional morphometric analysis of individual microglia and astrocytes in specific brain nuclei at different time points during the progression of heart failure. To this end, we used a well-established ischemic heart failure rat model. Morphometric studies were complemented with quantification of various pro-inflammatory cytokines and A1/A2 astrocyte markers via qPCR.

**Results:**

We report structural remodeling of central amygdala microglia and astrocytes during heart failure that affected cell volume, surface area, filament length, and glial branches, resulting overall in somatic swelling and deramification, indicative of a change in glial state. These changes occurred in a time-dependent manner, correlated with the severity of heart failure, and were delayed compared to changes in the hypothalamic paraventricular nucleus. Morphometric changes correlated with elevated mRNA levels of pro-inflammatory cytokines and markers of reactive A1-type astrocytes in the paraventricular nucleus and central amygdala during heart failure.

**Conclusion:**

We provide evidence that in addition to the previously described hypothalamic neuroinflammation implicated in sympathohumoral activation during heart failure, microglia, and astrocytes within the central amygdala also undergo structural remodeling indicative of glial shifts towards pro-inflammatory phenotypes. Thus, our studies suggest that neuroinflammation in the amygdala stands as a novel pathophysiological mechanism and potential therapeutic target that could be associated with emotional and cognitive deficits commonly observed at later stages during the course of heart failure.

## Background

According to the World Health Organization, cardiovascular diseases are the major cause of death globally (World Health Organization, 2017 [1];. The vast majority of cardiovascular disease-related deaths (85%) can be attributed to stroke and heart failure (HF), both having significant physical detrimental effects on the human body, including arrythmias, thromboembolisms, and paralysis among others [2]. Importantly, a growing body of clinical studies support a high degree of comorbidity between cardiovascular diseases and cognitive impairments and emotional distress [3–5]. In fact, 20–40% of all HF patients develop major depression and display anxiety levels significantly higher than the healthy population [3, 4, 6, 7], which usually appear later than the cardiovascular and autonomic-related symptoms [8]. These cognitive/mood disorders have also been observed in experimental animal models of HF, including the rat and mouse left coronary ligation model [9–11]. Still, the precise underlying neuronal substrates and mechanisms leading to mood disorders and cognitive impairments after HF remain largely unknown.

The paraventricular nucleus of the hypothalamus (PVN) is a key brain nucleus involved in the regulation of sympathetic outflow and cardiovascular control [12–15], playing thus a major role in bodily homeostasis [16–18]. These actions are mediated via direct innervation of sympathetic-related brainstem and spinal cord neurons [12–15]. There is also compelling evidence supporting a critical role for the PVN in the onset and maintenance of sympathohumoral activation during HF [19–23]. Importantly, neuroinflammation within the PVN has been described as a hallmark pathophysiological mechanism contributing to increased sympathetic outflow during this disease [24–27]. The PVN is also recognized as an important center in emotional regulation and harbors a variety of cells producing different neuropeptides [28–30]. In line with this, the PVN directly communicates with the lateral subdivision (CeL) of the central amygdala (CeA), which plays an important role in depression [31, 32], fear, and anxiety [33–35]. Oxytocin-synthesizing neurons in the PVN project long-range axonal terminals to the CeL, where the local release of oxytocin directly regulates anxiety levels [36, 37], fear memories, and fear extinction [38, 39]. Moreover, a recent study highlighted the pivotal role of oxytocin signaling in the CeA in mouse models of depression [40]. While neuroinflammation in the PVN of HF rats has been previously described [26, 27], it is at present unknown whether HF-induced neuroinflammation in other brain areas, such as the CeA, also occurs, and if so, how it temporally relates to the onset of neuroinflammation in the PVN.

Neuroinflammation is predominantly mediated by microglia, the immune cells that take up residence in the brain parenchyma [41, 42]. While classical markers such as increased microglial density, increased expression of ionized calcium-binding adapter molecule 1 (IBA1), and various cytokines are widely used to assess neuroinflammation, they provide only indirect information about detailed microglial morphology changes during this pathological process. This is critical because diverse microglial morphometric features are not only associated with diverse microglial functions [43, 44], but more importantly, they have been recently associated with different stages in the spatio-temporal progression of the neuroinflammation process [45]. Moreover, there is considerable microglial heterogeneity and brain region-specific differences in size, density, or pro-inflammatory stages [46]. Therefore, a detailed 3D analysis of microglia cell morphology could provide additional and critical insights into their role during different stages of the progression of the neuroinflammatory process. Such detailed analysis and quantification of microglial morphometry in the PVN and CeA under control and disease conditions, such as HF has, to the best of our knowledge, not been performed yet, and this constitutes a major goal of the present work. Given that neuroinflammation involves an intricate interplay between microglia and astrocytes [47–49], we were also interested to determine whether HF would alter astrocyte function and morphology as well.

To this end, we created a comprehensive glial morphometric profiler to perform a detailed quantitative analysis of microglial and astrocyte morphology in the PVN and CeA of HF rats in a time-dependent manner 8, 14, and 16 weeks (abbreviated as 8w, 14w, and 16w) after the onset of the disease. Moreover, we correlated these morphological parameters with the assessment of various classical markers of neuroinflammation via qPCR (IBA1, GFAP, TNF-α, IL-1β, and IL-6) as well as markers for astrocyte A1 (neurotoxic) and A2 neuroprotective phenotypes (Serping1, C3, Sphk1, and Tm4sf1) [49–52]. Our results highlight for the first time that in addition to the PVN, HF also induces a robust microglial/astrocyte cell remodeling as well as increased cytokine levels in the CeA, a critical forebrain region involved in emotion and cognition. Neuroinflammation-related changes in the CeA occurred with a delayed time course compared to those in the PVN, and in both cases, correlated with the severity of the disease, suggesting that neuroinflammation could potentially contribute to cognitive impairment and mood disorders observed at later stages of the disease in HF patients.

## Materials and methods

All performed experiments were approved by the Georgia State University Institutional Animal Care and Use Committee (IACUC) and carried out in agreement with the IACUC guidelines. At all time, animals had ad libitum access to food and water, and all efforts were made to minimize suffering and the numbers of animals used for this study.

### Animals

We used male Wistar rats (5–7 weeks old at surgery, 180–200 g, Envigo, Indianapolis, IN, USA) for all experiments (total *n* = 57, immunohistochemistry and IMARIS analysis 29; qPCR 28). Rats were housed in cages (2 per cage) under constant temperature (22 ± 2 °C) and humidity (55 ±5%) on a 12-h light/dark cycle (lights on 08:00–20:00).

### Heart failure surgery and echocardiography

HF was induced by coronary artery ligation as previously described [22]. In brief, animals were anaesthetized using 4% isoflurane and intubated for mechanical ventilation. To exteriorize the heart, we performed a left thoracotomy. The ligation was performed on the main diagonal branch of the left anterior descending coronary artery. Animals received buprenorphine SR-LAB (0.5 mg/kg, S.C.; ZooPharm, Windsor, CO, USA) before the surgical procedure to minimize post-surgical pain. Sham animals underwent the same procedure except the occlusion of the left coronary artery. Five weeks after the surgery, we performed transthoracic echocardiography (Vevo 3100 systems; Visual Sonics, Toronto, ON, Canada) under light isoflurane (2–3%) anesthesia. We obtained the left ventricle internal diameter and the left diameter of the ventricle posterior and anterior walls in the short-axis motion imaging mode to calculate the ejection fraction (EF). The myocardial infarct surgery typically results in a wide range of functional HF, as determined by the EF measurements. Rats with EF< 40% were considered as fully developed HF (32.48 ± 2.01% (*n* = 21), whereas those with EF > 50% were considered to express a mild form of HF (64.9 ± 2.1% (*n* = 5). The average EF in sham rats was 79.13 ± 1.57% (*n* = 21). Unless otherwise indicated (Fig. S4a–d), most studies compared sham and fully developed HF. Animals were used 8, 14, or 16 weeks after the HF surgery and allocated to the respective groups as indicated in the individual experiments. In addition, we used a naïve control group, which comprised rats that did not undergo any surgical procedure.

### Immunohistochemistry

Following pentobarbital-induced anesthesia (Euthasol, Virbac, ANADA #200-071, Fort Worth, TX, USA, pentobarbital, 80 mg/kg bw, i.p.), rats were first perfused at a speed of 20 mL/min with 0.01 M PBS (200 mL, 4 °C) through the left ventricle followed by 4% paraformaldehyde (PFA, in 0.3 M PBS, 200 mL, 4 °C), while the right atrium was opened with an incision. Brains were post-fixated for 24 h in 4% PFA at 4 °C and transferred into a 30% sucrose solution (in 0.01 M PBS) at 4 °C for 3–4 days. For immunohistochemistry, 40 μm slices were cut using a Leica Cryostat (CM3050 S), and brain slices were kept in 0.01 M PBS at 4 °C until used for staining. Brain slices containing the PVN (A/P, Bregma: − 1.1 mm to − 1.6 mm) and the CeA (A/P, Bregma: − 2.0 mm to − 2.8 mm) were blocked with 5% Normal Horse Serum in 0.01 M PBS for 1 h at room temperature. After a 15-min washing in 0.01 M PBS, brain slices were incubated for 24 h in 0.01 M PBS, 0.1% Triton-X, 0.04% NaN_3_ containing 1:1000 of anti-IBA1 (polyclonal rabbit, Wako, 019-19741, Lot: CAK1997), 1:1000 anti-glutamine synthetase (monoclonal mouse, Merck Milipore, MAB 302, clone GS-6), or anti-GFAP (goat polyclonal, abcam, ab53554) at room temperature. Following the 15-min washing in 0.01M PBS, sections were incubated in 0.01 M PBS, 0.1% Triton-X, 0.04% NaN_3_ with 1:500 Alexa Fluor 488/594-conjugated donkey anti-rabbit/goat/mouse (Jackson ImmunoResearch, 711-585-152, 705-585-147, 715-545-151) for 4 h at RT. Brain slices were washed again for 15 min in 0.01 M PBS and mounted using anti-fade mounting medium (Vectashield H-1500).

### Confocal microscopy and 3D IMARIS analysis

For the 3D reconstruction of microglia, we took Z-stack images (50 μm depth, 1 μm steps, × 40 magnification) of PVN, CeA (lateral and medial subdivision of the CeA, but not the capsular subdivision) and somatosensory cortex 1 barrel field (S1BF) using a Zeiss LSM 780 confocal microscope (1024 × 1024 pixel, 16-bit depth, pixel size 0.63 μm, zoom 0.7). Images for all brain regions (2 per hemisphere, 6 sections in total) were obtained from the center of the respective structures using the third ventricle, stria terminalis, and lateral ventricle as landmarks for the PVN, CeA, and somatosensory cortex 1 barrel field, respectively. All images were obtained from the center of the respective brain structures to minimize overlap with neighboring brain regions. Raw czi files were used for further analysis using IMARIS software (Version 9.31, Oxford Instruments). First, IMARIS was used to reconstruct the microglial surface using the following custom settings: surfaces Detail 0.700 μm (smooth); thresholding background subtraction (local contrast), diameter of largest sphere, which fits into the object: 2.00; color: base, diffusion transparency: 65%. After surface reconstruction, we used the filter function to remove unspecific background signals: filter: volume max–400 μm^3^. All microglia with incomplete somata (cut by either the *x*, *y*, or *z* plane) were manually removed and not included in further analysis. In addition, reconstructed entities that were clearly not microglia (i.e., filaments without soma) were also manually removed. Fused microglia that were falsely recognized as one entity by the software were manually separated using the cut function or entirely removed from the sample if a separation was not feasible. The “filter/area function” was used to remove small microglial segments that occurred during manual. After deletion of all background signals, the “mask all” function was used to create the final surface reconstruction. Next, the surface reconstruction was used as the template for the filament reconstruction using the following custom settings: detect new starting points: largest diameter 7.00 μm, seed points 0.300 μm; remove seed points around starting points: diameter of sphere regions: 15 μm. Seed points were corrected for (either placed in or removed from the center of the somata) manually if the IMARIS algorithm placed them incorrectly. All surface and filament parameters were exported into separate Excel files and used for data analysis. For all quantifications, we used 6–8 40× z-stacks per animal (2 z-stacks per brain hemisphere). On average, the reconstruction of a single z-stack took 15 min using a computer suited for IMARIS analysis (Intel Core i7 8700 at 3.2 GHz, 64 GB RAM, x-64-bit, Windows 10 Enterprise), which included the manual removal of microglia. All images used for analysis were taken with the same confocal settings (pinhole, laser intensity, digital gain, and digital offset). Image processing, three-dimensional reconstruction, and data analysis were performed in a blind manner in regards to the experimental conditions.

### Sholl analysis

Sholl analysis was performed using IMARIS in the filament reconstruction mode, and individual data sets were exported into separate Excel files for further analysis. We defined a threshold for microglial complexity (peak number for Sholl intersections per microglia): < 10.0 intersections (deramified), > 9.9 (ramified), and we calculated microglial complexity based on these defined thresholds. For the total number of Sholl intersections, we added together all the intersections (from each individual sphere) per individual microglia.

### Reverse transcription polymerase chain reaction (RT-PCR) and quantitative real time PCR (qPCR)

RNA extraction and isolation were performed using the miRNAeasy Mini kit (Qiagen, cat. no. 217004) and the QIAzol Lysis Reagent (Qiagen, mat. no. 1023537). Briefly, 250-μm-thick tissue sections were made in cryostat (− 20 °C, Leica, CM3050S) and punches from the PVN (2 mm punches, 4 punches per animal for pooled qPCR, 6 punches for individual samples), CeA (1 mm punches, 8 punches per animal, for pooled qPCR, 10 punches for individual samples) were collected and kept in dry ice until the RNA extraction procedure. We performed initially a qualitative approach (Fig. S5), in which punches (*N* = 4 and 8 and per animal for PVN and CeA, respectively, *n* = 5 animals per group) were pooled together within the respective group, and triplicate technical replicates were performed. Subsequently, a quantitative approach was used, in which punches (*N* = 6 and 10 per animal for PVN and CeA, respectively, *n* = 5 animals per group) were used individually without pooling. RNA concentration was measured using NanoDrop One (Thermo Scientific) and was in the range of 115–220 ng/μl prior to cDNA synthesis. cDNA synthesis was performed using the iScript^TM^ gDNA Clear cDNA Synthesis Kit (BIO RAD, cat. no. 1725035) and the SimpliAmp Thermal Cycler (applied biosystems, Thermo Fisher Scientific) following the manufacturer’s protocol. qPCR was conducted using the following 10× QuantiTect primers (diluted in 1.1 mL TE pH 8.0, final concentration 200 nM) purchased from Qiagen: IBA1 (QT01591751), GFAP, IL-1β (QT00181657), IL-6 (QT00182896), Serping1 (QT01607326), C3 (QT00187159), Tm4sf1 (QT01588034), Sphk1 (QT00182035), TNF-α (QT00182896), and β-Actin (QT00193473, used as the reference gene). All individual qPCR reactions (brain region, primer, and condition) were triplicated.

### Statistical analysis

All statistical analyses were performed using GraphPad Prism 8 (GraphPad Software, CA, USA). The significance of differences was determined using Student’s *t* test or two-way ANOVA for two-group comparisons followed by Tukey post hoc test, as indicated throughout the text. For all statistical tests regarding microglial parameters, we calculated the mean value for the respective parameter for each animal so that the final “*n*” was the number of animals tested and used these values for statistical analysis. For the generation and comparison of Sholl distribution curves and to analyze the shift in microglial populations, we used individual microglia values, but still performed the respective statistical tests with the average value of each animal. For the quantitative qPCR (Fig. 6), we performed measurements using individual samples (each sample was tested twice and averaged) and performed a one-sample *t* test using ± 1 as the hypothetical mean. Results are expressed as mean ± standard error of the mean (SEM). If not mentioned otherwise, statistical comparisons were performed between HF groups and their respective sham groups. Results were considered statistically significant if *p* < 0.05 and are presented as * for *p* < 0.05, ** for *p* < 0.01 and *** for *p* < 0.0001 in the respective figures.

## Results

### Development and validation of a comprehensive 3D morphometric microglia profiler

To obtain a detailed 3D quantitative morphometric analyses of glia cells in HF (Fig. S1a–d), we applied our novel glial profiler based on the IMARIS software to an established model of HF [23, 53, 54] at different time points post-surgery (Fig. S1e, f). Briefly, we took 50 μm z-stack confocal images (16-bit, 1 μm steps, × 40 objective) of brain slices stained with IBA1 and exported czi files for further analysis in IMARIS. Incompletely stained microglia were not included into our analysis (detailed description of IMARIS-based analysis can be found in “Materials and methods” section). This method allows an unbiased high throughput analysis of various cellular features including surface area, cell volume, or filament length, and is a universally applicable tool for morphometric analysis of different cell types under various conditions. To assess whether the sham surgery itself resulted in microglia morphometric alterations over time, we perfused naïve (20 weeks old, *n* = 4) and sham rats (8, 14, and 16 weeks post-surgery, *n* = 4/group). We stained brain sections containing the PVN and the CeA with IBA1 and analyzed microglial profiles using IMARIS. We did not observe any significant changes in the PVN or CeA of sham rats in any of these parameters either when compared to naïve rats (20w) or as a function of time after the sham surgical procedure (8 weeks, 14 weeks, or 16 weeks, Fig. S2a–d). Therefore, we decided to pool together the three sham groups for further analysis.

### HF-induced time-dependent morphometric microglial changes in the PVN are already evident at 8 weeks post-surgery

**Fig. 1:**
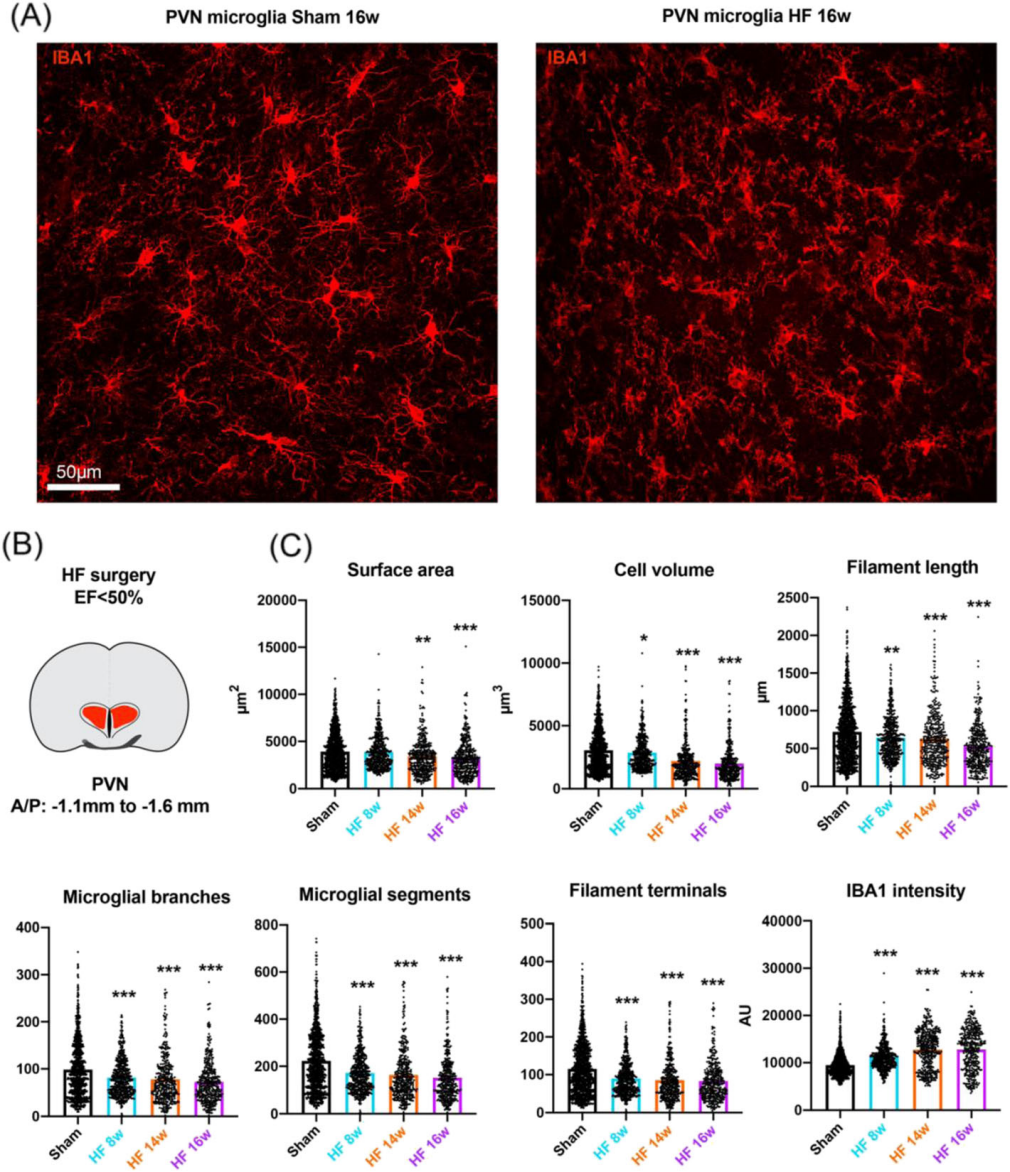
HF-induced morphometric changes in PVN microglia. **a** Representative confocal images show IBA1-stained microglia in the PVN of HF and sham rats 16 weeks post-surgery. **b** Brain scheme shows the topographic location of PVN microglia that have been used for the morphometric assessment. The red area within the PVN (heart-shaped nucleus) highlights the fraction of the nucleus where pictures were taken. **c** Dot-plot graphs show the individual values of PVN microglia for surface area, cell volume, filament length, microglial branches, microglial segments, filament terminals, and IBA 1 intensity for sham rats (*N* = 1135 cells from 12 rats, pooled) and HF rats at 8, 14, and 16 weeks post-surgery (*N* = 378 cells from 4 rats, *N* = 407 cells from 4 rats and *N* = 399 cells from 4 rats, respectively). *p < 0.05, **p < 0.01, and ***p < 0.0001 vs. respective sham, one-way ANOVA followed by Tukey’s post hoc test

To study HF-induced microglial changes in the PVN and CeA, we assessed microglial surface area, cell volume, filament length, microglial branches, microglial segments, filament terminals, and IBA1 intensity in sham and HF rats at 8, 14, and 16 weeks after the surgery (*n* = 4/group) (Fig. 1). We found significant morphometric microglial changes including a decrease in surface area, cell volume, and filament length already at 8 weeks post-surgery, which progressed as a function of time (Fig. 1).

To rule out that HF-induced changes in microglia morphology was a diffuse phenomenon affecting functionally unrelated brain regions, we chose the primary somatosensory cortex (barrel field, S1BF, Fig. S3a–c) as a control region that was located on the same brain slices to warrant comparable IBA1 exposure and tissue quality and analyzed IBA1-labeled microglia in this structure. We did not observe any significant microglial changes within this region, suggesting that, at least at this stage, cortical brain areas do not display significant changes in microglial morphology.

Lastly, to determine whether the observed microglial changes were dependent on the severity of the disease, we included an additional control group of rats that underwent the myocardial infarction surgery (and used at 16 weeks post-surgery), but that developed only a mild form of functional heart failure, indicated by an EF > 50% (mean EF 64.9 ± 2.1%, *n* = 5). We found that a mild functional HF was not associated with any significant changes of microglial morphology (Fig. S4a–c). Moreover, this additional group allowed us to perform a correlative analysis of the degree of microglia changes as a function of the severity of HF (i.e., as a function of EF value). As summarized in Figure S4d, we found a strong and significant correlation for cell volume, filament length, and number of microglial branches with %EF, supporting the notion that morphometric changes in microglia during HF are dependent on the severity of the disease.

Taken together, these findings indicate that HF-induced microglial morphological changes in the PVN are evident as early as 8 weeks after the myocardial infarction, they progress in a time-dependent manner, and correlate with the severity of the disease.

### HF-induced delayed morphometric microglial changes in the CeA correlated with the severity of the disease

**Fig. 2:**
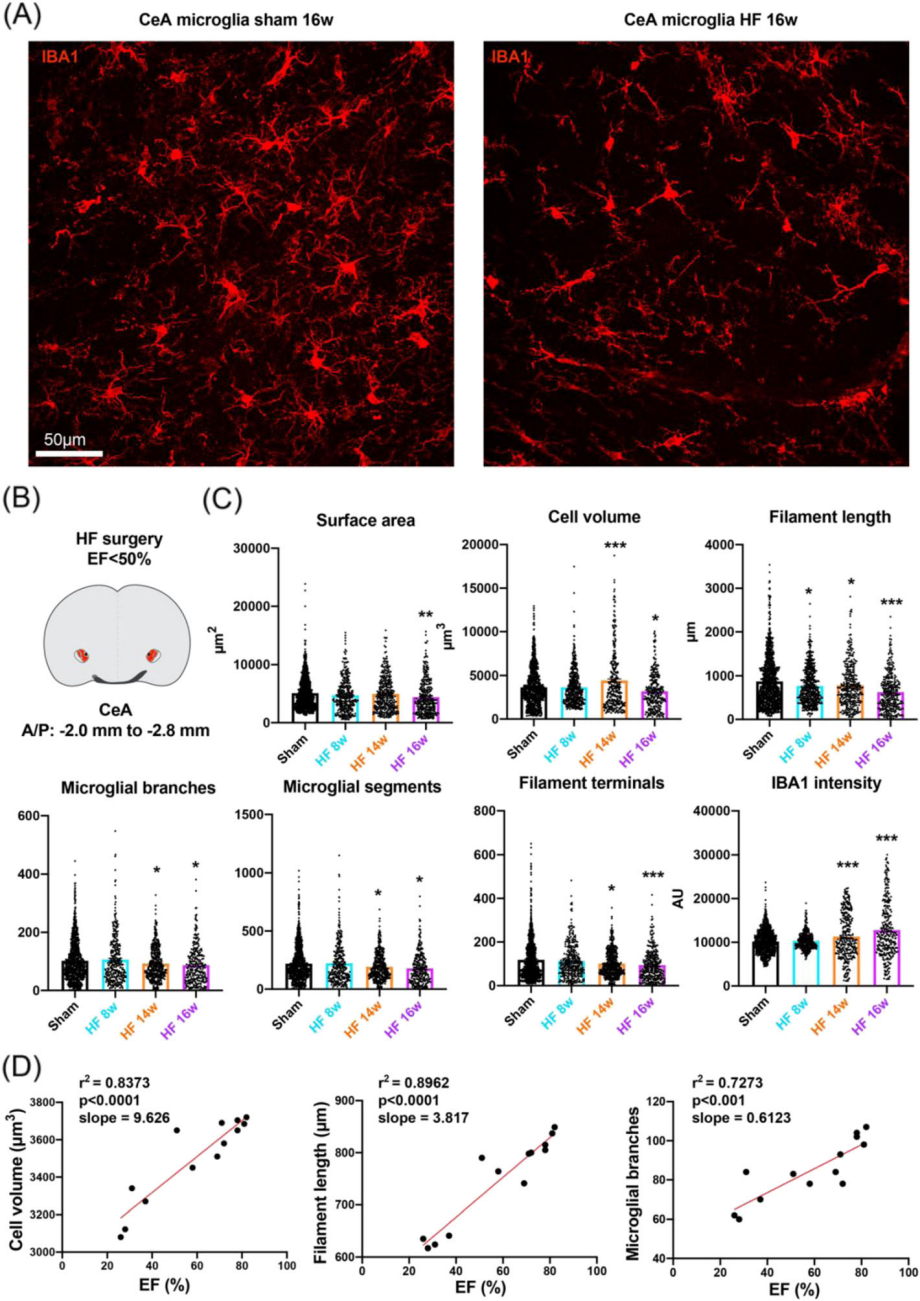
HF-induced morphometric changes in CeA microglia. **a** Representative confocal images show IBA1-stained microglia in the PVN of HF and sham rats 16 weeks post-surgery. **b** Brain scheme shows the topographic location of CeA microglia that have been used for the morphometric assessment. The red dotted line highlights the CeA. **c** Dot-plot graphs show the individual values of CeA microglia for surface area, cell volume, filament length, microglial branches, microglial segments, filament terminals, and IBA 1 intensity for sham rats (*N* = 1070 cells from 12 rats, pooled) and HF rats at 8, 14, and 16 weeks post-surgery (*N* = 363 cells from 4 rats, *N* = 355 cells from 4 rats and *N* = 332 cells from 4 rats, respectively). *p < 0.05, **p < 0.01, and ***p < 0.0001 vs. respective sham, one-way ANOVA followed by Tukey’s post hoc test. **d** Plot graphs depicting cell volume, filament length, and number of microglial branches as a function of %EF values combining sham (*n* = 4), HF rats (EF < 50%, *n* = 4) and mild HF rats (EF > 50%, *n* = 5). *R*^2^ and *p* values were obtained following a Pearson correlation analysis

In stark contrast to the PVN, except for a slight decrease in filament length, we did not detect any significant changes in CeA microglia morphology 8 weeks after the myocardial infarction (Fig. 2). However, significant changes in microglial morphometric parameters emerged at 14 weeks (Fig. 2c), which continued to progress at week 16. It is important to note that while we observed an initial increase of microglial cell volume at 14 weeks post-surgery in CeA, at 16 weeks post-surgery, the cell volume was significantly decreased compared to the sham group. In line with what we observed in the PVN, microglia morphometric parameters were not changed in rats displaying a mild form of HF (i.e., EF > 50%; *n* = 5, see Table 1). Importantly, we also found key CeA microglia morphometric endpoints to strongly correlate with the severity of the disease (Fig. 2d). Taken together, these results indicate that CeA microglia undergo morphometric changes during HF, which occurred with a delayed time course compared to PVN microglia and which also correlated to the severity of the disease.

**Table 1:** Summary of microglial morphometric parameters of sham and HF rats for the PVN and CeA

Brain region	Group	Cell volume (μm^3^)	Surface area (μm^2^)	Filament length (μm)	Microglial branches	Microglial segments	Filament terminals	IBA1 intensity (AU)
PVN	Sham 8 weeks *N* = 381 cells *n* = 4 animals	3157 ± 112	4048 ± 99	745 ± 21	104 ± 3	219 ± 7	116 ± 4	9449 ± 123
	Sham 14 weeks *N* = 365 cells *n* = 4 animals	3098 ± 119	4100 ± 107	736 ± 18	102± 4	224 ± 8	118 ± 6	9385 ± 135
	Sham 16 weeks *N* = 389 cells *n* = 4 animals	3181 ± 122	4068 ± 81	761 ± 25	100 ± 5	216 ± 7	117 ± 6	9522 ± 139
	HF 8 weeks *N* = 378 cells *n* = 4 animals	2838 ± 58	3868 ± 75	642 ± 12	82 ± 2	171 ± 5	90 ± 3	11138 ± 118
	HF 14 weeks *N* = 407 cells *n* = 4 animals	2212 ± 74	3424 ± 64	629 ± 35	78 ± 2	163 ± 7	85 ± 3	12764 ± 202
	HF 16 weeks *N* = 399 cells *n* = 4 animals	2012 ± 69	3233 ± 59	524 ± 39	72 ± 3	150 ± 5	83 ± 4	12783 ± 231
	HF 16 weeks EF > 50% *N* = 435 cells *n* = 5 animals	3234 ± 128	3988 ± 90	741 ± 27	97 ± 6	216 ± 8	115 ± 9	9433 ± 146
CeA	Sham 8 weeks *N* = 377 cells *n* = 4 animals	3588 ± 137	4885 ± 119	841 ± 28	109 ± 6	219 ± 7	114 ± 3	10134 ± 137
	Sham 14 weeks *N* = 339 cells *n* = 4 animals	3644 ± 122	4966 ± 131	865 ± 31	102 ± 2	214 ± 5	116 ± 4	10073 ± 144
	Sham 16 weeks *N* = 354 cells *n* = 4 animals	3691 ± 123	4904 ± 127	833 ± 24	104 ± 5	216 ± 7	114 ± 3	10285 ± 180
	HF 8 weeks *N* = 363 cells *n* = 4 animals	3634 ± 119	4700 ± 124	765 ± 29	106 ± 4	222 ± 7	112 ± 3	10344 ± 176
	HF 14 weeks *N* = 355 cells *n* = 4 animals	4418 ± 187	4964 ± 144	781 ± 28	92 ± 4	192 ± 8	100 ± 3	11314 ± 277
	HF 16 weeks *N* = 332 cells *n* = 4 animals	3180 ± 115	4395 ± 163	623 ± 24	89 ± 3	179 ± 6	94 ± 4	12779 ± 357
	HF 16 weeks EF > 50% *N* = 384 cells *n* = 5 animals	3540 ± 132	4798 ± 141	835 ± 56	94 ± 4	202 ± 7	110 ± 4	11322 ± 290
S1BF	Sham 14 weeks *N* = 139 cells *n* = 4 animals	4577 ± 138	6008 ± 234	967 ± 31	125 ± 5	261 ± 8	135 ± 7	10539 ± 183
	HF 14 weeks *N* = 162 cells *n* = 4 animals	4257 ± 130	5664 ± 224	957 ± 31	126 ± 4	264 ± 8	138 ± 8	10647 ± 171

Table shows individual values (mean ± SEM) of all analyzed microglial parameters, *N* = number of analyzed microglia, *n* = number of animals per group, *CeA* = central amygdala, *PVN* = paraventricular nucleus, *S1BF* = somatosensory cortex barrel field 1

### HF resulted in microglial deramification and somatic swelling in both the PVN and CeA

**Fig. 3:**
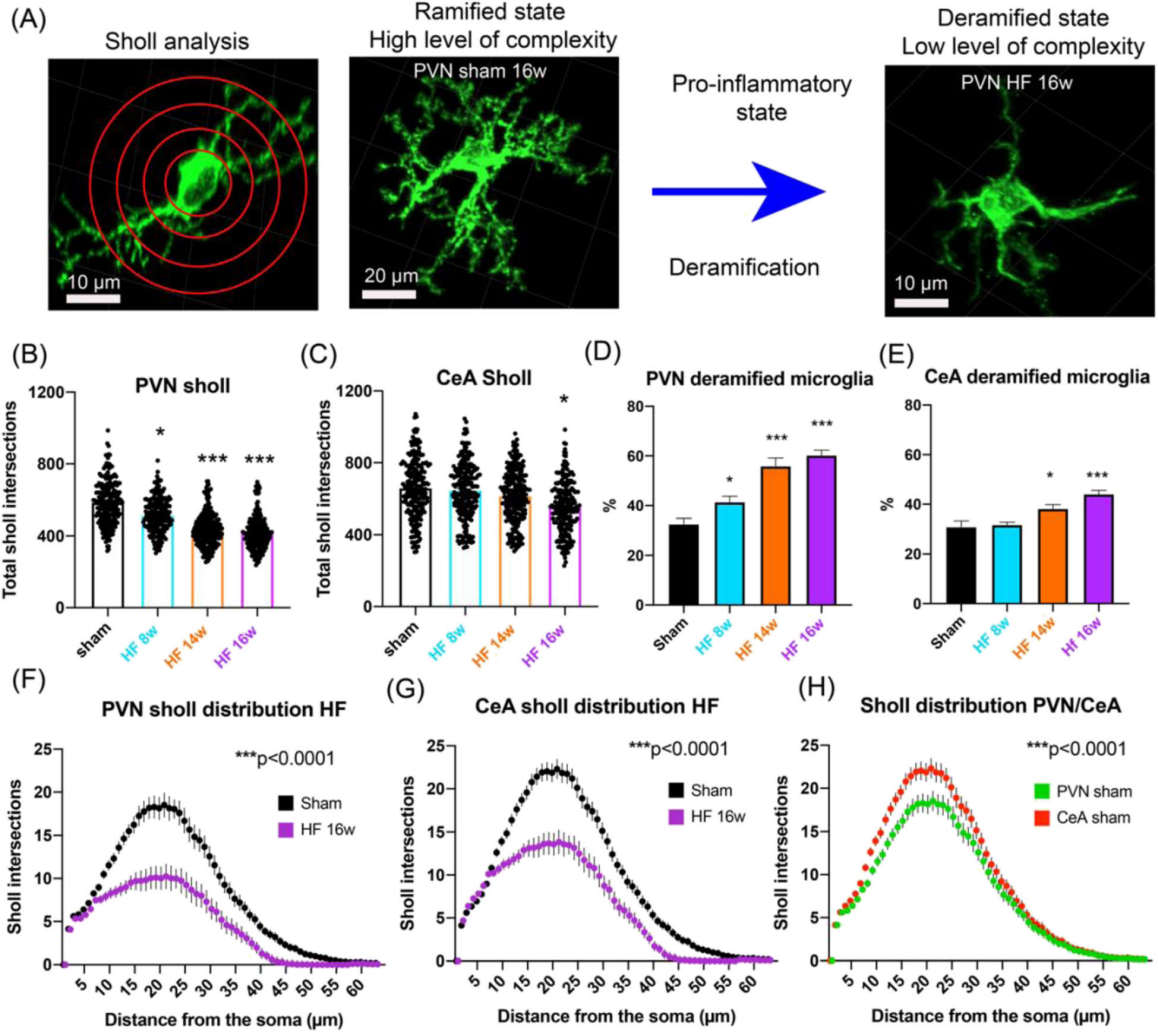
HF-induced deramification and increased the proportion of pro-inflammatory microglia in the PVN and CeA. **a** Scheme depicts the HF-induced transition from ramified to deramified microglia. Red circles depict superimposed spheres centered around microglia somata used for Sholl analysis. **b**, **c** Bar graphs show the mean number of total of Sholl intersections for PVN and CeA microglia in sham rats (*n* = 12, pooled) and HF rats at 8, 14, and 16 weeks post-surgery (*n* = 4/group). **d**, **e** Bar graphs show the mean proportion of deramified microglia in the PVN and CeA in sham rats (*n* = 12, pooled) and HF rats at 8, 14, and 16 weeks post-surgery (*n* = 4/group). **f**, **g** Mean distribution plots of the number of Sholl intersections as a function of the distance from the microglial soma for sham and HF rats 16 weeks post-surgery (*n* = 4/group). **h** Distribution plot comparing Sholl intersections as a function of the distance from the microglial soma for PVN and CeA microglia (*n* = 12/group, pooled). *p < 0.05, **p < 0.01, and ***p < 0.0001 vs. respective sham, two-way ANOVA, or one-way ANOVA followed by Tukey’s post hoc test

As stated above, pro-inflammatory microglia have been demonstrated to undergo deramification, a process where microglia retract their processes, lose microglial complexity, and release inflammatory cytokines [43, 55]. Thus, to further investigate microglial cell morphometric changes during HF in the PVN and CeA, we performed a Sholl analysis of individually 3D-reconstructed microglial cells in each experimental group. To this end, we superimposed spheres of increasing radius (1 μm increase in radius per step, Fig. 3a) starting at the center of the soma and measured the number of process intersections that each sphere encountered. We found that in the PVN (Fig. 3b), microglia displayed a significant loss of complexity (indicated by a significantly reduced average number of total Sholl intersections) that occurred in a time-dependent manner with changes being already detectable 8 weeks post-surgery, and becoming progressively more deramified at 14 and 16 weeks post-surgery (Fig. 3b, 11.1%, *p* = 0.008, 17.1%, *p* < 0.0001 and 30.8%, *p* < 0.0001 at 8, 14, and 16 weeks, respectively, one-way ANOVA, *F* = 36.3, *p* < 0.0001). In the CeA (Fig. 3c), we did not observe such changes at 8 or 14 weeks post-surgery. However, 16 weeks post-surgery, microglia in the CeA displayed significant deramification (16.41%, *p* = 0.036, one-way ANOVA, *F* = 7.42, *p* < 0.0001). To quantify the degree of overall microglia deramification, we first determined the peak number of Sholl intersections (i.e., the highest numeric value of a sphere intersecting with a microglial process) per individual microglial cell, which in our entire sampled microglia cell population ranged from 0 to 64 (the higher the number, the more ramified the microglial structure). Using a semi-arbitrary and conservative threshold of < 10 to categorize a microglial cell as pro-inflammatory, we found 32.5% deramified microglia in the PVN (Fig. 3d) and 30.9% in the CeA (Fig. 3e) of sham rats 14 weeks post-surgery. In the PVN HF rats, we found a significant increase in the number of deramified microglia 8 weeks post-surgery (41.4% *p* = 0.0125, one-way ANOVA, *F* = 25.70, *p* < 0.0001), which had further increased at 14 weeks (55.9%, *p* < 0.0001) and 16 weeks (60.25%, *p* < 0.0001). In the CeA of HF rats, a significant increase in the number of deramified microglia only became evident at 14 weeks (38.2%, *p* = 0.0201), increasing further at 16 weeks (44.0%, *p* < 0.0001, one-way ANOVA, *F* = 41.34, *p* < 0.0001).

Finally, we compared the overall Sholl distribution curves between sham and HF in the PVN and the CeA at 16 weeks post-surgery (Fig. 3f, g). We found significant differences for both the PVN (two-way ANOVA, group: *F* (1, 56) = 96.48, *p* < 0.0001, Fig. 3f) and the CeA (two-way ANOVA, group: *F* (1, 56) = 83.77, *p* < 0.0001, Fig. 3g). In the PVN, the mean peak number of Sholl intersections was reached at 21 μm distance from the soma for both sham and HF rats, and the mean number of intersections at the peak was significantly different between those groups (sham 18.5 ± 1.2 intersections vs. HF 16 weeks: 10.2 ± 0.9 intersections, *p* < 0.0001). In the CeA, this peak was reached at 22 μm for the sham group and 21 μm for the HF group, and was also significantly different between the two groups (sham 21.9 ± 1.6 intersections vs. HF 16 weeks 13.9 ± 1.3 intersections, *p* < 0.0001). Interestingly, under control conditions, microglial cells in the CeA were significantly more complex than in the PVN (two-way ANOVA, group: *F* (1, 56) = 74.13, *p* < 0.0001, Fig. 3h), highlighting the morphological heterogeneity among microglia within different brain regions. Taken together, these findings indicate that microglia in both PVN and CeA undergo deramification during HF.

**Fig. 4:**
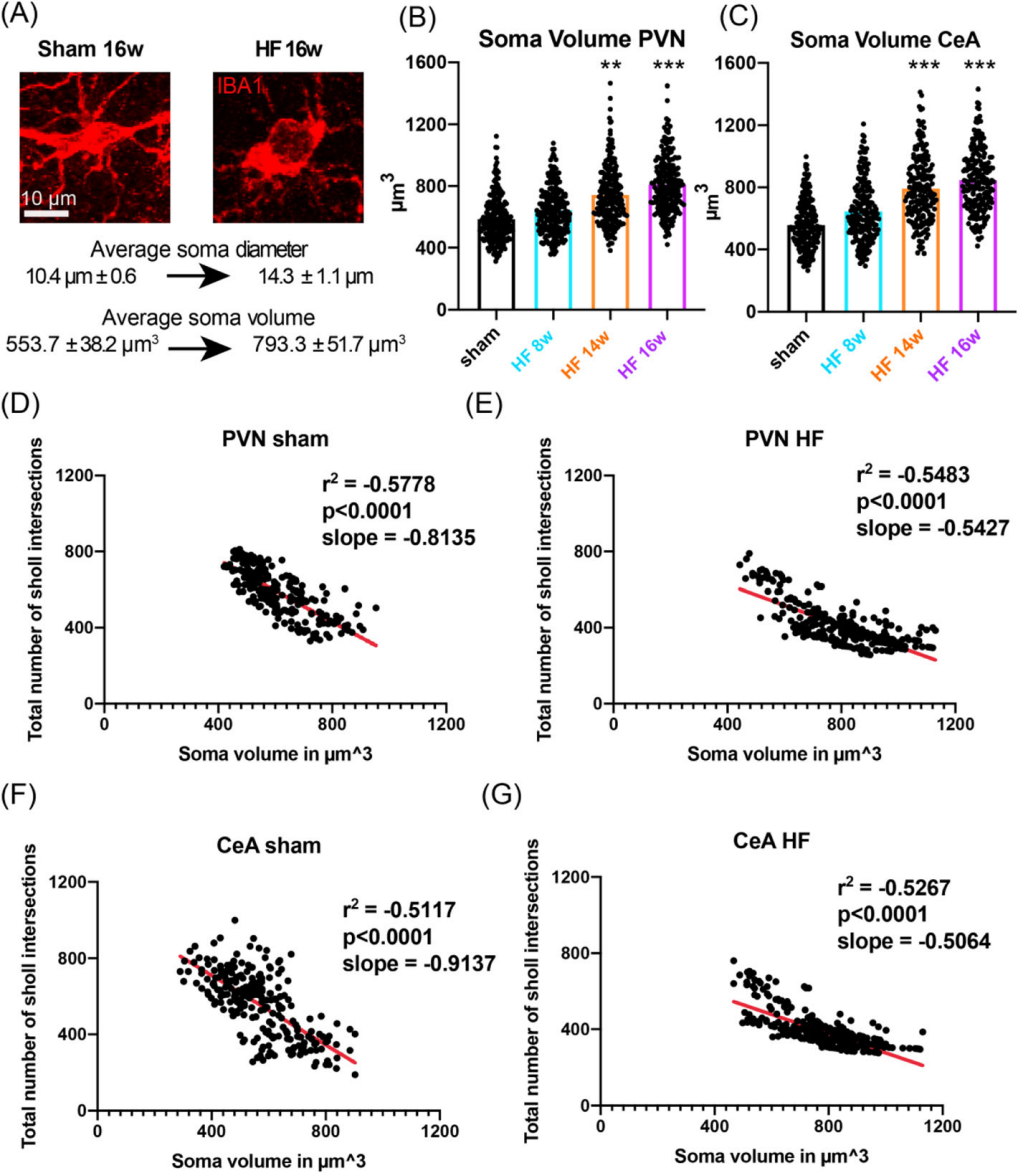
HF-induced somatic swelling of PVN and CeA microglia is correlated with microglial deramification. **a** Confocal images show a representative example of HF-induced microglial swelling. **b**, **c** Bar graphs show the mean microglia somata volume in the PVN and CeA in sham rats (*n* = 12, pooled) and HF rats at 8, 14, and 16 weeks post-surgery (*n* = 4/group). **d**–**g** Plots showing the total number of Sholl intersections as a function of soma volume for individual microglial cells in the PVN of sham (**d**) and HF (**e**) rats and in the CeA of sham (**f**) and HF (**g**) rats. Red lines represent best-fit lines assuming a non-linear relationship between the total number of Sholl intersections and soma volume. **p < 0.01 and ***p < 0.0001 vs. respective sham, one-way ANOVA followed by Tukey’s post hoc test or Pearson correlation

A recent study highlighted that in addition to deramification, pro-inflammatory microglia display somatic swelling [57], a process thought to coincide with the release of pro-inflammatory cytokines, especially in neurodegeneration [58]. Thus, to investigate whether HF resulted in somatic microglia swelling, we calculated the somatic volume of individual microglial cells. We found that the average microglial soma volume of sham rats 14 weeks post-surgery was 554.3 ± 21 μm^3^ for the PVN and 529.0 ± 41 μm^3^ for the CeA (Fig. 4a–c). We found a time-dependent increase in somatic volume of PVN microglia 28.2%, *p* = 0.0025 and 43.1%, *p* < 0.0001 at 14 and 16 weeks, respectively, compared to the respective sham group, one-way ANOVA, *F* = 12.48, *p* < 0.0001. In the CeA, this effect was even more pronounced time-dependent 41.3%, *p* < 0.0001 and 51.2%, *p* < 0.0001 increases in somatic volume at 14 and 16 weeks, respectively, compared to the respective sham group, one-way ANOVA, *F* = 20.37, *p* < 0.0001.

To determine whether somatic swelling and microglial deramification were correlated processes or whether they occurred independently in separate microglial subpopulations, we performed a correlative analysis between these two parameters (Fig. 4d–g). We found that both in sham and HF rats, the increase in somatic volume and the degree of microglial filament ramification were negatively correlated both in the PVN and CeA (*p* < 0.0001 for all cases). Interestingly, we observed an apparent decrease in the slope of the best-fit non-linear regression line (Fig. 4d–g, red lines) in HF compared to sham rats, in both brain regions, which might be indicative of the less polarized (i.e., more homogenous) microglial cell population spectrum in HF rats, resulting from the shift towards a pro-inflammatory microglial stage

### HF induced a morphological A1 astrocyte phenotype in both the PVN and CeA

**Fig 5:**
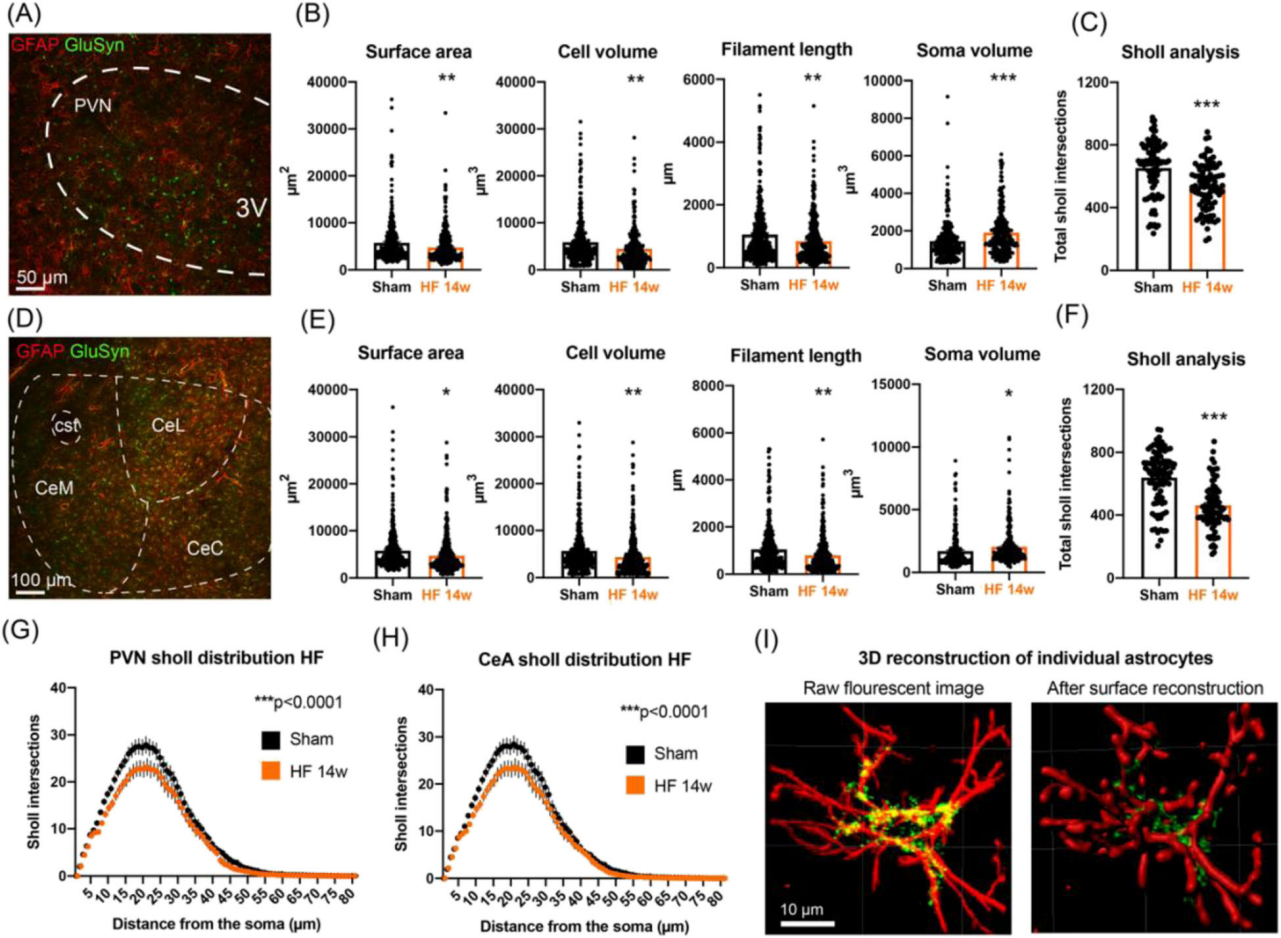
HF-induced morphometric changes in PVN and CeA astrocytes. **a** Representative confocal image showing PVN astrocytes in a sham rat stained with GFAP (red) and GluSyn (green). **b** Dot-plot graphs show the individual values of PVN microglia for surface area, cell volume, filament length, and IBA1 intensity in sham (*n* = 4) and HF rats 14 weeks post-surgery (*n* = 4). **c** Bar graph shows the mean number of total sholl intersections in PVN astrocytes in sham (*n* = 4) and HF rats 14 weeks post-surgery (*n* = 4). **d** Representative confocal image showing CeA astrocytes stained with GFAP (red) and GluSyn (green) in a sham rat. **e** Dot-plot graph show the individual values of CeA microglia for surface area, cell volume, filament length, and IBA1 intensity in sham (*n* = 4) and HF rats 14 weeks post-surgery (*n* = 4). **f** Bar graph shows the mean number of total Sholl intersections in CeA astrocytes in sham (*n* = 4) and HF rats 14 weeks post-surgery (*n* = 4). **g**, **h** Mean distribution plots of the number of Sholl intersections as a function of the distance from the microglial soma for sham and HF rats 14 weeks post-surgery (*n* = 4/group). **i** Confocal images show a single PVN astrocyte from a sham animal before and after surface reconstruction with IMARIS. *p < 0.05, **p < 0.01, and ***p < 0.0001 vs. respective sham, Student’s *t* test, or two-way ANOVA

During injury and disease, microglia and astrocytes display intricate interactions that may lead either to neuronal survival or neuronal loss. A recent study showed that “activated” (i.e., pro-inflammatory) microglia induce a neurotoxic A1 subtype of astrocytes, which secrete a currently unknown neurotoxin that results in neuronal cell death [49]. A1 astrocytes can be discriminated from the neuroprotective A2 astrocytes not only by the upregulation of genetic markers [48–50, 59], but also by distinct morphological changes, which appear to be similar to those of microglia during neuroinflammation [60]. Given that neuroinflammation-induced changes in astrocytes seem to generally follow those observed in microglia [48, 49], we chose to investigate astrocytic changes at 14 weeks post-surgery. To this end, we performed 3D IMARIS analysis of astrocytes that were immunohistochemically identified by their expression of both GFAP and glutamine synthetase (GluSyn) (Fig. 5a, d), which preferentially stain for astrocyte processes and soma, respectively (see Fig. 5i). In HF rats, we found significant morphological changes in both PVN and CeA astrocytes 14 weeks post-surgery (*n* = 4 rats/group) that included a decrease in surface area (17.2%, *p* = 0.0062 and 19.1%, *p* = 0.022, for PVN and CeA, respectively), cell volume (24.4%, *p* = 0.001 and 23.2%, *p* = 0.001, for PVN and CeA, respectively), filament length (19.4%, *p* = 0.0052 and 24.6%, *p* = 0.0033, for PVN and CeA, respectively), and an increase in soma volume (30.8%, *p* < 0.0001 and 18.8%, *p* = 0.026, for PVN and CeA, respectively, Fig. 5b, e). Furthermore, we found that astrocytes of HF rats displayed a significant loss of process complexity, as shown by a significant decrease in the total number of Sholl intersections per astrocyte both in the PVN (18.0%) and the CeA (27.8%, *p* < 0.0001 in both cases, Fig. 5c, f). In addition, the Sholl distribution analysis revealed significant changes in astrocyte complexity 14 weeks post-surgery for both the PVN (two-way ANOVA, group: *F* (1, 61) = 84.32, *p* < 0.0001, Fig. 5g) and CeA (two-way ANOVA, group: *F* (1, 56) = 70.92, *p* < 0.0001, Fig. 5h). Figure 5j shows an isolated PVN astrocyte of a HF rat and subsequent surface reconstruction via IMARIS.

### HF induced expression of genes associated with neuroinflammation and A1 astrocyte phenotype in both PVN and CeA

To determine whether the microglial/astrocyte morphometric changes observed in HF rats also corresponded with a genetic profile associated with neuroinflammation and/or a shift to an A1 astrocyte phenotype, we performed qPCR of mRNA transcripts of various genes classically associated with neuroinflammation, as well as several A2 and A1 astrocyte-related genes. We first performed a qualitative study in which we pooled samples from micropunches obtained from the PVN and CeA of sham and HF rats at the same time points (8 weeks and 14 weeks) at which the morphometric studies were performed and analyzed mRNA levels of IBA1, GFAP, cytokines (TNF-α, IL-1β, and IL-6), A1 astrocyte markers (Serping1 and C3), and A2 astrocyte markers (Tm4sf1 and Sphk1) (Fig. S5a–d). We found several neuroinflammation-related mRNAs such as cytokines or A1 astrocyte markers to be upregulated, while others, including A2 astrocyte markers or GFAP were downregulated.

**Fig. 6:**
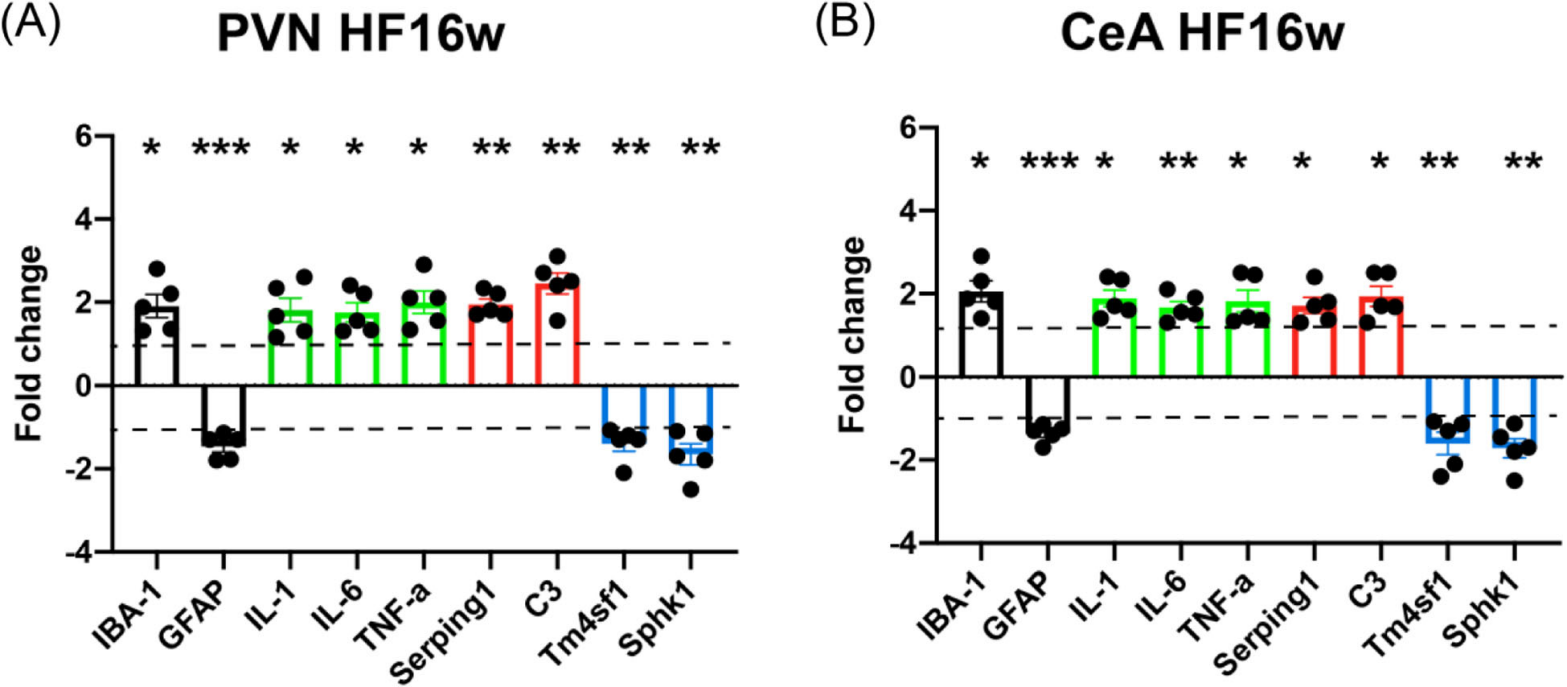
HF-induced changes in neuroinflammation-associated microglia and astrocyte marker mRNA. **a** Bar graphs show the mean fold change in mRNA transcript levels in HF rats compared to sham rats in the PVN (**a**) and the CeA (**b**). All graphs depict the fold changes of mRNA levels in HF group compared to their respective shams. Green bars: cytokines, red bars: a1 astrocyte markers, blue bars: a2 astrocyte markers. Dashed lines indicate the hypothetical mean (+ 1/− 1) for the one-sample *t* test. Each dot represents an individual animal in the respective group. *p < 0.05, **p < 0.01, and ***p < 0.0001 vs. respective controls, one sample *t* test

Next, to quantitatively assess changes in mRNA levels, we repeated the qPCR experiment, this time using individual samples (*n* = 5 per group). For this quantitative assessment of HF-related changes in mRNA levels, we used rats 16 weeks post-surgery to be able to directly compare those results with the latest time point of our IHC-based analysis. The results were largely consistent with the qualitative studies, as we found statistically significant differences in all tested genes between sham and HF animals for both the PVN and the CeA (Fig. 6).

## Discussion

### Heart failure-induced neuroinflammation and its physiological and behavioral correlates

Cardiovascular diseases including stroke and HF are the leading cause of deaths worldwide, and affected individuals suffer from severe physiological and psychological impairments [1]. While compounds like angiotensin-converting enzyme inhibitors, angiotensin receptor blockers, mineralocorticoid receptor antagonists, or β-blockers have been successfully used to treat the physiological symptoms of HF patients with reduced EF [62], little to no treatment is available for HF-induced mood and anxiety disorders. In fact, recent studies suggest that the classical use of selective serotonin reuptake inhibitors may not be an efficient way to relieve depression symptoms in HF patients [56, 63]. Importantly, neuroinflammation and oxidative stress in the PVN and the rostro-lateral medulla have been reported in rats with HF and neurogenic hypertension [26, 64, 65]. It is well established that inflammation and activation of the renin-angiotensin system drastically increases sympathetic drive, thereby creating a vicious circle with debilitating effects on affected individuals [66]. Moreover, intracerebroventricular infusions of minocycline, an anti-inflammatory antibiotic that inhibits microglia cell function, resulted in significant attenuation of mean arterial pressure and cardiac hypertrophy [64]. These changes were accompanied by a reduction in the total number of pro-inflammatory microglia as well as reduced mRNA levels for IL-1β, IL-6, and TNF-α [64]. Taken together, these previous studies pinpoint neuroinflammation, particularly proinflammatory microglial cells within the PVN, as a key underlying pathophysiological mechanism contributing to the sympatho-humoral and cardiovascular complications associated with HF [24–27]. Given that the PVN is a key regulatory structure involved in the integration of behavioral, cardiovascular, and neuroendocrine homeostatic responses [67–69], and is richly interconnected with a plethora of brain regions [33, 70–72], including the amygdala, it is reasonable to speculate that neuroinflammation could also contribute to mood and anxiety disorders associated with HF. In fact, PVN-amygdala connectivity is well established [33, 70–72] and the release of various PVN-synthesized neuropeptides within the amygdala has been shown to directly modulate aggression, fear, and depression [38, 39, 73]. However, to the best of our knowledge, no previous study demonstrated that HF is associated with a pro-inflammatory microglial profile consisting of somatic swelling and a shift from a ramified to a deramified state.

### Microglial deramification in the paraventricular nucleus and central amygdala in heart failure rats

Microglia, the resident immune cells of the brain parenchyma, are among the first responding cells during injury, cell death, or unwanted intruders that might access the brain due to a compromised blood-brain barrier [41, 43, 44, 55, 74]. Microglia represent a major component in the immune response and participate in the neuroinflammatory response largely via the release pro-inflammatory cytokines [43, 55]. During neuroinflammation, microglia undergo a morphological transition from a highly ramified to a deramified state, retracting their fine processes while simultaneously undergoing somatic hypertrophy [43, 44, 75]. Although it seems evident that a comprehensive knowledge of microglial deramification is of paramount importance, very little is known about the precise series of events that ultimately lead to deramification, highlighting the need for novel tools that allow a detailed morphometric analysis of microglial remodeling during the progression of a neuroinflammatory process. While other laboratories employed comparable approaches for the reconstruction of microglia under various conditions using different analytical tools [76–78], these approaches are very time-consuming (80 min or more per z-stack), and could result in over- and/or undersampling given that fused (microglia recognized as one entity by the software) or cut microglia (somata or processes cut by either the *x*, *y*, or *z* axis) are included in the analysis. These limitations are largely overcome by our glial profiler based on the IMARIS software which allows for an unbiased, rapid, and fully automated reconstruction and morphometric analysis of individually identified microglial cell. Moreover, our profiler identifies and either separates or eliminates incomplete or fused microglial profiles. While this is not the first study to implement IMARIS analysis for three-dimensional reconstruction of microglia [79, 80], our in-depth analysis of microglia morphology and their morphometric transition to a pro-inflammatory state in the context of the progression and severity of a disease model represents, to the best of our knowledge, an unprecedented approach. With our 3D profiler, we were able to detect time-dependent microglia morphological changes in HF rats including filament shortening, deramification, and somatic swelling in both the PVN and CeA. These changes are widely accepted as indicators of a pro-inflammatory microglial state [43, 44, 55, 57, 76]. We found that microglial cell remodeling in the PVN and CeA worsened over time (Figs. 1 and 2), which is highlighted in part by a progressive increase in the number of deramified microglia (Fig. 3) and a shift of the microglial population towards a less complex microglial phenotype. Although the retraction of microglial processes and somatic swelling was highly correlated (Fig. 4), we found an initial increase in total cell volume in the CeA (14 weeks, Fig. 2) suggesting that somatic swelling might precede the deramification. Another important finding of the current study is that processes make up the majority of microglial volume (5–6× more volume in processes than in somata, see Figs. 1, 2 and S2-4), providing an explanation for the overall decrease in cell volume despite the observed increase in somatic volume following HF. Our data also suggest that HF-induced microglial cell changes were not representative of a diffused phenomenon globally affecting the brain, given that a non-related region, the S1BF (Fig. S3) was not affected, at least at this time point of the disease. Finally, it is important to note that we found significant differences in cell volume, surface area, and IBA1 intensity when we compared microglial cells between the PVN and the CeA in sham rats (Fig. 3). These results are in line with previous studies performed both in rodents and humans supporting a high degree of heterogeneity of microglial phenotypes that might depend on age, brain region, and pathophysiological condition [44, 61, 81].

### Region-specific differences and time-dependency of heart failure-induced neuroinflammation

We found that microglial cell remodeling in the PVN is present already at 8 weeks post-surgery, while microglial changes in the CeA started to unfold around the 14-week mark. While our study demonstrated for the first time HF-induced microglial cell remodeling in the CeA, a major finding was that these changes occurred in a delayed manner compared to the PVN. These time-dependent microglial structural changes were consistent and correlated as well with qPCR analysis of pro-inflammatory markers, such as TNF-α, IL-1β, and IL-6, which in the PVN were already significantly elevated at 8 weeks, while in the CeA most markers became elevated at 14 weeks post-surgery. These results further support the previously established relationship between microglial cell morphological changes with the release of pro-inflammatory cytokines [43, 44].

As stated above, a large body of evidence supports a key role for the PVN in the onset and maintenance of cardiovascular dysregulation in HF [12, 19–23]. Thus, the time-dependent differences in microglial deramification and pro-inflammatory markers between the PVN and the amygdala are in line with the fact that sympathohumoral and cardiovascular manifestations in HF precede the mood and cognitive deficits associated with this disease [62, 82, 83]. HF-induced cognitive impairments in both rats and mice has been previously described [84, 85] and while it is well established that the PVN is a main neuronal substrate contributing to mood regulation, our studies provide an indirect evidence that neuroinflammation within the amygdala may constitute a neuronal substrate contributing to HF-induced mood deficits. A recent study showed that the microglia inhibitor minocycline improved depression-like behavior in HF rats [86], but the authors performed oral delivery of minocycline so that the precise site of action within the brain underlying this improvement could not to be determined. Given the neuroprotective effects of minocycline in acute ischemic stroke [87], as well as a reduction of neuroinflammation in a model of neurodegeneration [88], this microglia inhibitor could be a promising therapeutic candidate for HF-triggered, neuroinflammation-induced cognitive and mood disorders, albeit the respective brain regions underlying those impairments need to be identified. Thus, further studies are clearly necessary to demonstrate that neuroinflammation within the CeA does in fact contribute to HF-induced depression-like behavioral changes and that a reduction of neuroinflammation within the CeA can alleviate some or most of the associated symptoms.

The mechanisms underlying the time course differences between the HF-induced neuroinflammation in PVN and CeA could be manifold and not mutually exclusive. Based on the tightly coupled interaction between the PVN and the amygdala [33, 70–72], neuroinflammation in the amygdala could be triggered by the exaggerated neuronal activity in the PVN reported in HF [22, 23, 89, 90]. This is in line with previous studies showing that aberrant neuronal activity could result in the release of damage-associated molecules, leading to microglia activation [91, 92]. Recent studies also support the ability of inflammatory cytokines to diffuse in the extracellular space [93–95]. Thus, the delay for neuroinflammation onset in the amygdala could be due to the slow lateral diffusion of cytokines initially originating in the PVN. Finally, the delay may also be due to differential responsiveness of PVN and amygdala microglial cell populations to the same noxious stimulus (i.e., HF-associated hypoxia) and/or differences in blood-brain barrier integrity, between the PVN and CeA during disease states, which could result in region-specific permeability differences, as we recently showed to be the case during hypertension [96]. Lastly, it is important to highlight that we found a strong correlation between key microglia morphometric parameters (both in PVN and CeA) and the degree of functional heart failure, as measured by %EF, suggesting that microglia morphometric changes were also dependent on the severity of the disease. These findings are in line with human studies supporting that the progression of both cognitive decline [97] and depression [98] might directly be linked to the ejection fraction/severity of the disease. Clearly, future studies are warranted to address the precise mechanisms underlying the differential and region-specific time course of pro-inflammatory microglial states during HF.

### Morphological changes in PVN and CeA astrocytes and shift towards the A1 astrocyte phenotype

The intricate interaction between microglia and astrocytes during neuroinflammation is well established [60, 99, 100]. Microglial cells release various pro-inflammatory molecules upon injury [101] or as a result of the general immune response [102], which in turn triggers specific astrocytic responses. Astrocytes can release orosomucoid-2 to inhibit pro-inflammatory microglia, block the chemokine receptor type 5 of the microglial membrane, downregulate the inflammatory response, or further promote neuroinflammation and neurodegeneration [100]. This bidirectional interaction allows an efficient and tailored response to various potentially harmful threats that the brain might be exposed to. Recent studies proposed the classification of astrocytes into two dynamic categories: A1 (pro-inflammatory, neurotoxic) and A2 (anti-inflammatory, neuroprotective) [48–50]. During the transition from the A2 to A1 phenotype, astrocytes are thought to undergo morphometric changes comparable to those observed in microglia. Indeed, and similar to what we found with microglial cells, our results demonstrated significant morphological changes in both PVN and CeA astrocytes during HF. Importantly, these morphometric changes were accompanied by the upregulation of A1-specific (Serping1, C3) and downregulation of A2-specific (Tm4sf1 and Sphk1) astrocyte markers. Taken together, these results indicate that HF promotes a shift in astrocyte phenotype from A2 to A1 both in the PVN and CeA, contributing thus along microglial cells to the overall neuroinflammatory state in this disease. Interestingly, we found a significantly decreased GFAP mRNA levels in HF rats of both PVN and CeA. While these findings might seem contrary to the general notion that GFAP protein levels are increased in A1 astrocytes, recent studies suggest that GFAP might not be the best marker for astrocyte reactivity [48] and highlight that the relationship between reactive astrocytes and GFAP levels might have been oversimplified [103]. It is important to note that while we report here significant changes in glial morphology and cytokine levels, it remains unclear whether these changes translate into altered glial function, and how they might ultimately affect the pro-inflammatory state within the respective brain regions. Moreover, it is well known that microglia are actively involved in multiple functional roles, including phagocytosis, synaptic remodeling, clearing apoptotic neuronal debris, and the shaping of neuronal networks among others [104–110]. It seems plausible that HF-induced, pro-inflammatory changes interfere with these vital functions, which might further affect proper neuronal/network function beyond the pro-inflammatory state itself. However, how exactly somatic swelling and retraction of microglial processes impairs these important functions needs to be further explored.

It is important to note that we exclusively used male rats in our study and given that sex differences in microglia are well established and have been described for several brain regions (111, 112), it is plausible that microglia respond differently to heart failure in female rats. Finally, most published studies in the field using the congestive HF rodent model, including the present one, were performed in young adult rats (6–10 weeks) [24, 27, 113–115]. This is a caveat that needs to be taken into consideration, given that the incidence of HF in humans is higher in the elderly population.

Taken together, our findings suggest that HF-induced neuroinflammation not only alters the microglial environment in affected brain regions, but rather has far-reaching consequences affecting astrocytes and potentially even neurons due to astrocyte-induced neurotoxicity [48, 49]. Additional studies assessing neuronal apoptosis are needed to determine whether the HF-induced A1 astrocyte phenotype in the CeA becomes neurotoxic.

## Conclusion

In summary, in the present study, we implemented a novel and improved quantitative morphometric imaging approach to assess precise microglial and astrocyte structural remodeling in different brain regions during the progression of heart failure. We provide evidence that in addition to the previously described neuroinflammation observed in the hypothalamic PVN, known to be implicated in sympathohumoral activation during this disease, microglia and astrocytes within the central amygdala (CeA) also undergo structural remodeling. These structural changes are indicative of glial shift towards a pro-inflammatory microglial and a potentially neurotoxic astrocyte phenotype, a finding supported also by an increased expression of pro-inflammatory cytokines and A1/2 astrocyte markers. Importantly, our results show that neuroinflammation in the CeA manifested with a delayed time course with respect to the PVN and was dependent on the severity of the disease. Taken together, our studies support the idea that neuroinflammation in the amygdala may contribute to emotional and cognitive deficits commonly observed at later stages during the course of HF, standing thus as a potential novel target for alleviating comorbid mood disorders in this prevalent disease [3, 5, 116].

## Supplementary information

**Additional file 1:.** Figure S1: Heart failure surgery, qPCR and three-dimensional reconstruction (a) representative image showing the echocardiographic assessment left ventricular cardiac function in a sham and HF rats, (b) timetable depicting morphometric assessments and qPCR at 8-, 14- and 16- weeks post-surgery, (c, d) representative confocal images showing IBA1-labeled microglia (red) in the PVN and CeA and the topographic delineation of the nuclei, (e) schematic depiction of the sequential steps used for the 3D morphometric reconstruction and assessment of microglia (IBA1, green), (f) confocal images show the step-by-step process for three-dimensional reconstruction of a single microglia (IBA1, red). For image processing, colors were changed from red to green in IMARIS for better visibility and increased precision during manual editing of reconstructed microglia. Note the merging of red (raw fluorescence) and green (surface reconstruction) channels in the top panel, 3V: third ventricle, AW: anterior wall, CeC: central amygdala capsular division, CeL: central amygdala lateral division, CeM: central amygdala medial division, cst: commissural stria terminalis, LVID, d and LVID, s: left ventricle internal dimensions in diastole and systole, PVN: paraventricular nucleus, PW: posterior wall. Figure S2: Sham surgery per se does not alter the morphology of PVN or CeA microglia (a) brain scheme shows the topographic location of PVN microglia that have been used for the morphometric assessment. The red area within the PVN (heart-shaped nucleus) highlights the fraction of the nucleus where pictures were taken, (b) dot-plot graphs show the individual values of PVN microglia for surface area, cell volume, filament length and IBA 1 intensity for the non-surgery control (N=357 cells from 4 rats), and sham rats at 8, 14 and 16 weeks post-surgery (N=381 cells from 4 rats, N= 365 cells from 4 rats and N=389 cells from 4 rats, respectively), one-way ANOVA was used to analyze the data followed by Tukey post-hoc test, (c) brain scheme shows the topographic location of CeA microglia that have been used for the morphometric assessment. The red area within the CeA highlights the divisions where pictures were taken, (d) dot-plot graphs show the individual values of CeA microglia for surface area, cell volume, filament length and IBA 1 intensity for the non-surgery control (N=297 cells from 4 rats), and sham rats at 8, 14 and 16 weeks post-surgery (N=377 cells from 4 rats, N=339 cells from 4 rats and N=354 cells from 4 rats, respectively). Figure S3: HF does not induce morphometric changes in somatosensory cortex 1 barrel field microglia (a) representative confocal images show IBA1-stained microglia in the S1BF of HF and sham rats 16 weeks post-surgery, (b) brain scheme shows the topographic location of S1BF microglia that have been used for the morphometric assessment. The red area highlights the S1BF, (c) dot-plot graphs show the individual values of S1BF microglia for surface area, cell volume, filament length, microglial branches, microglial segments, filament terminals and IBA 1 intensity for sham rats (n=4) and HF rats (n= 4). One-way ANOVA was used to analyze the data. Figure S4: Morphological changes in microglia are not observed in rats with mild level of HF (a) representative confocal images show IBA1-stained microglia in the PVN of sham and rats that underwent heart failure surgery, but did not develop severe HF (‘mild HF’), indicated by an ejection fraction greater than 50% (EF>50%), (b) brain scheme shows the topographic location of PVN microglia that have been used for the morphometric assessment. The red area highlights the PVN, (c) dot-plot graphs show the individual values of PVN microglia for surface area, cell volume, filament length, microglial branches, microglial segments, filament terminals and IBA 1 intensity for sham rats (n=4) and mild HF rats (n=5). One-way ANOVA was used to analyze the data, (d) plot graphs depicting cell volume, filament length and number of microglial branches as a function of %EF values combining sham (n=4), HF rats (EF<50%, n=4) and mild HF rats (EF>50%, n=5). R2 and p values were obtained following a Pearson correlation analysis. Figure S5: HF-induced changes in neuroinflammation-associated microglia and astrocyte marker mRNA levels (a-f) bar graphs show the mean fold change in mRNA transcript levels in HF rats compared to sham rats in the PVN (a, b) and the CeA (c, d). All graphs depict the fold changes of mRNA levels in HF group compared to their respective shams. Green bars: cytokines, red bars: a1 astrocyte markers, blue bars: a2 astrocyte markers. Dashed lines indicate the hypothetical mean (+1/-1) for the one-sample t-test. Each dot represents the average value of the pooled samples and the measurements were performed in triplicates. *p<0.05, **p<0.01 and ***p<0.0001 vs. respective controls, one sample t-test.

## Data Availability

The datasets used and/or analyzed in the current study are available from the corresponding author upon reasonable request.

## Abbreviations

C3: Complement component 3
CeA: Central amygdala
CeL: Lateral subdivision of the central amygdala
GFAP: Glial fibrillary acidic protein
GluSyn: Glutamine synthetase
HF: Heart failure
IL: Interleukin
Serping1: C1-inhibitor gene
Sphk1: Sphingosine kinase 1
Tm4sf1: Transmembrane 4 L6 family member 1
TNF-α: Tumor necrosis factor α
