# Correction to: Three-dimensional morphometric analysisreveals time-dependent structural changesin microglia and astrocytes in the centralamygdala and hypothalamicparaventricular nucleus of heart failure rats

**DOI:** 10.1186/s12974-020-02035-5

**Published:** 2020-11-22

**Authors:** Ferdinand Althammer, Hildebrando Candido Ferreira-Neto, Myurajan Rubaharan, Ranjan K. Roy, Atit A. Patel, Anne Murphy, Daniel N. Cox, Javier E. Stern

**Affiliations:** 1 Center for Neuroinflammation and Cardiometabolic Diseases, Georgia StateUniversity, Atlanta, USA; 2 Neuroscience Institute, Georgia State Universit, Atlanta, USA

**Correction to: J Neuroinflammation (2020) 17:221**

**https://doi.org/10.1186/s12974-020-01892-4**

Following publication of the original article [1], the authors noticed that Prof. Anne Murphy was mistakenly omitted from the manuscript authorship. Prof. Anne Murphy needs to be inserted as 6th author on this article. The original article has been updated.
