# Supplementary material for: Three-dimensional morphometric analysis reveals time-dependent structural changes in microglia and astrocytes in the central amygdala and hypothalamic paraventricular nucleus of heart failure rats
Source: J Neuroinflammation. 2020 Jul 23;17:221. doi: 10.1186/s12974-020-01892-4 (PMC7379770; doi:10.1186/s12974-020-01892-4)
Supplement: Supplementary file 1 — Additional file 1:. Figure S1: Heart failure surgery, qPCR and three-dimensional reconstruction (a) representative image showing the echocardiographic assessment left ventricular cardiac function in a sham and HF rats, (b) timetable depicting morphometric assessments and qPCR at 8-, 14- and 16- weeks post-surgery, (c, d) representative confocal images showing IBA1-labeled microglia (red) in the PVN and CeA and the topographic delineation of the nuclei, (e) schematic depiction of the sequential steps used for the 3D morphometric reconstruction and assessment of microglia (IBA1, green), (f) confocal images show the step-by-step process for three-dimensional reconstruction of a single microglia (IBA1, red). For image processing, colors were changed from red to green in IMARIS for better visibility and increased precision during manual editing of reconstructed microglia. Note the merging of red (raw fluorescence) and green (surface reconstruction) channels in the top panel, 3V: third ventricle, AW: anterior wall, CeC: central amygdala capsular division, CeL: central amygdala lateral division, CeM: central amygdala medial division, cst: commissural stria terminalis, LVID, d and LVID, s: left ventricle internal dimensions in diastole and systole, PVN: paraventricular nucleus, PW: posterior wall. Figure S2: Sham surgery per se does not alter the morphology of PVN or CeA microglia (a) brain scheme shows the topographic location of PVN microglia that have been used for the morphometric assessment. The red area within the PVN (heart-shaped nucleus) highlights the fraction of the nucleus where pictures were taken, (b) dot-plot graphs show the individual values of PVN microglia for surface area, cell volume, filament length and IBA 1 intensity for the non-surgery control (N=357 cells from 4 rats), and sham rats at 8, 14 and 16 weeks post-surgery (N=381 cells from 4 rats, N= 365 cells from 4 rats and N=389 cells from 4 rats, respectively), one-way ANOVA was used to analyze the d [file 12974_2020_1892_MOESM1_ESM.pdf]

Three-dimensional morphometric analysis reveals time-dependent structural changes in microglia and astrocytes in the central amygdala and hypothalamic paraventricular nucleus of heart failure rats

Ferdinand Althammer, Hildebrando Candido Ferreira-Neto, Myurajan Rubaharan, Ranjan Kumer Roy, Atit A. Patel, Daniel N. Cox and Javier E. Stern

Supplementary Materials

Figure S1

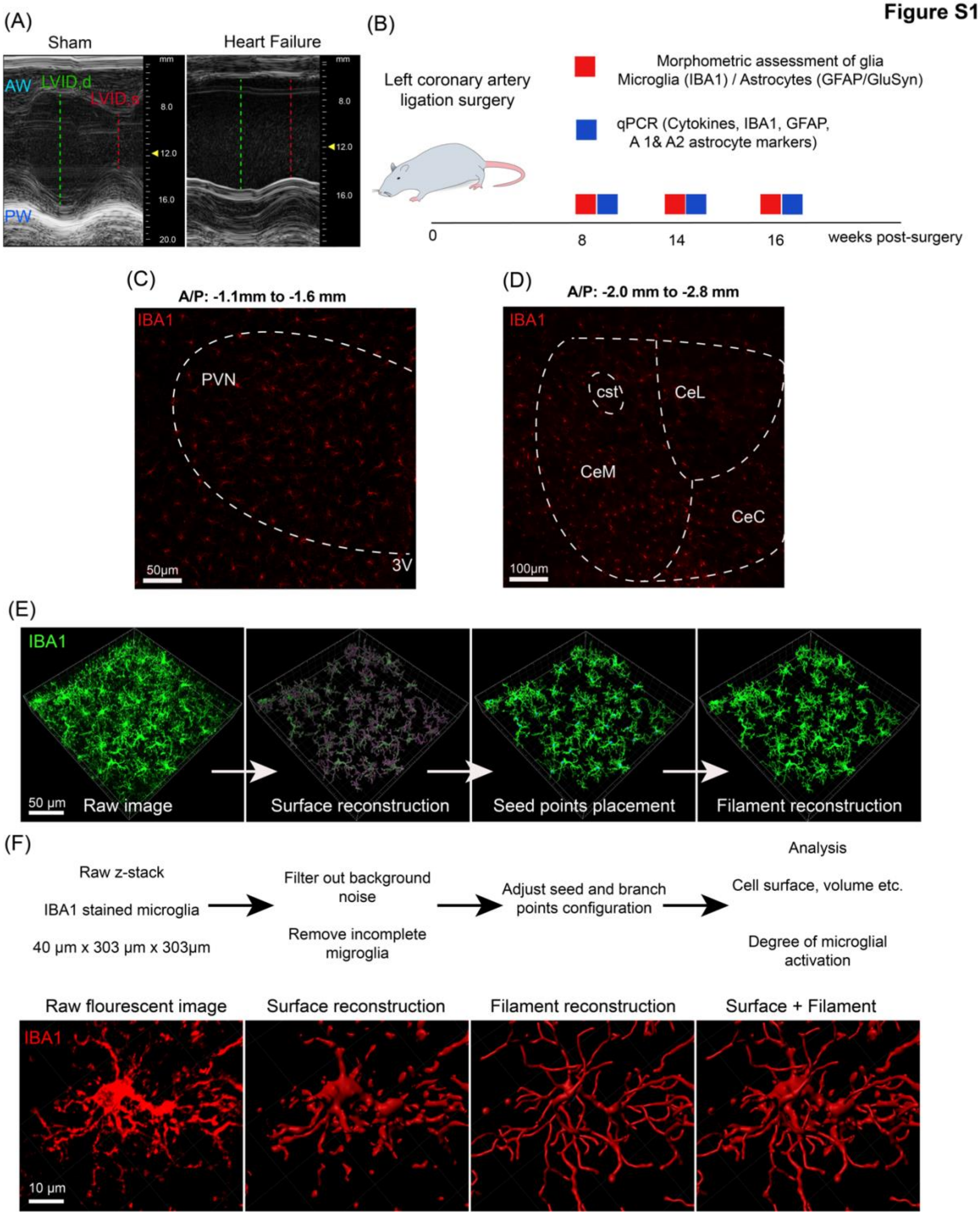

(A)
Sham
Heart Failure
AW
LVID;d
LVID;s
PW
mm
8.0
12.0
16.0
20.0
(B)
Left coronary artery ligation surgery
Morphometric assessment of glia
Microglia (IBA1) / Astrocytes (GFAP/GluSyn)
qPCR (Cytokines, IBA1, GFAP, A 1& A2 astrocyte markers)
0
8
14
16
weeks post-surgery
(C)
A/P: -1.1mm to -1.6 mm
IBA1
PVN
3V
50μm
(D)
A/P: -2.0 mm to -2.8 mm
IBA1
cst
CeL
CeM
CeC
100μm
(E)
IBA1
50 μm
Raw image
Surface reconstruction
Seed points placement
Filament reconstruction
(F)
Raw z-stack
IBA1 stained microglia
40 μm x 303 μm x 303μm
Filter out background noise
Remove incomplete migroglia
Adjust seed and branch points configuration
Analysis
Cell surface, volume etc.
Degree of microglial activation
Raw flourescent image
Surface reconstruction
Filament reconstruction
Surface + Filament
IBA1
10 μm

Figure S1: Heart failure surgery, qPCR and three-dimensional reconstruction (a) representative image showing the echocardiographic assessment left ventricular cardiac function in a sham and HF rats, (b) timetable depicting morphometric assessments and qPCR at 8-, 14- and 16- weeks post-surgery, (c, d) representative confocal images showing IBA1-labeled microglia (red) in the PVN and CeA and the topographic delineation of the nuclei, (e) schematic depiction of the sequential steps used for the 3D morphometric reconstruction and assessment of microglia (IBA1, green), (f) confocal images show the step-by-step process for three-dimensional reconstruction of a single microglia (IBA1, red). For image processing, colors were changed from red to green in IMARIS for better visibility and increased precision during manual editing of reconstructed microglia. Note the merging of red (raw fluorescence) and green (surface reconstruction) channels in the top panel, 3V: third ventricle, AW: anterior wall, CeC: central amygdala capsular division, CeL: central amygdala lateral division, CeM: central amygdala medial division, cst: commissural stria terminalis, LVID, d and LVID, s: left ventricle internal dimensions in diastole and systole, PVN: paraventricular nucleus, PW: posterior wall.

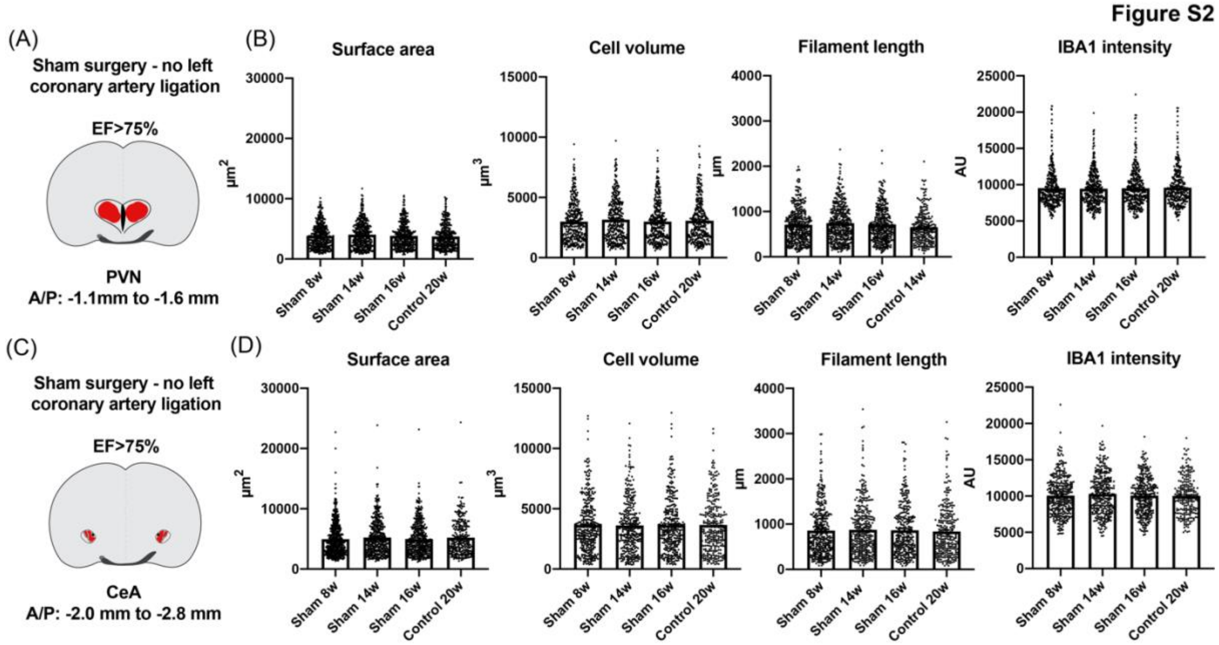

Figure S2: Sham surgery per se does not alter the morphology of PVN or CeA microglia (a) brain scheme shows the topographic location of PVN microglia that have been used for the morphometric assessment. The red area within the PVN (heart-shaped nucleus) highlights the fraction of the nucleus where pictures were taken, (b) dot-plot graphs show the individual values of PVN microglia for surface area, cell volume, filament length and IBA 1 intensity for the non-surgery control (N=357 cells from 4 rats), and sham rats at 8, 14 and 16 weeks post-surgery (N=381 cells from 4 rats, N= 365 cells from 4 rats and N=389 cells from 4 rats, respectively), one-way ANOVA was used to analyze the data followed by Tukey post-hoc test, (c) brain scheme shows the topographic location of CeA microglia that have been used for the morphometric assessment. The red area within the CeA highlights the divisions where pictures were taken, (d) dot-plot graphs show the individual values of CeA microglia for surface area, cell volume, filament length and IBA 1 intensity for the non-surgery control (N=297 cells from 4 rats), and sham rats at 8, 14 and 16 weeks post-surgery (N=377 cells from 4 rats, N=339 cells from 4 rats and N=354 cells from 4 rats, respectively).

Figure S3

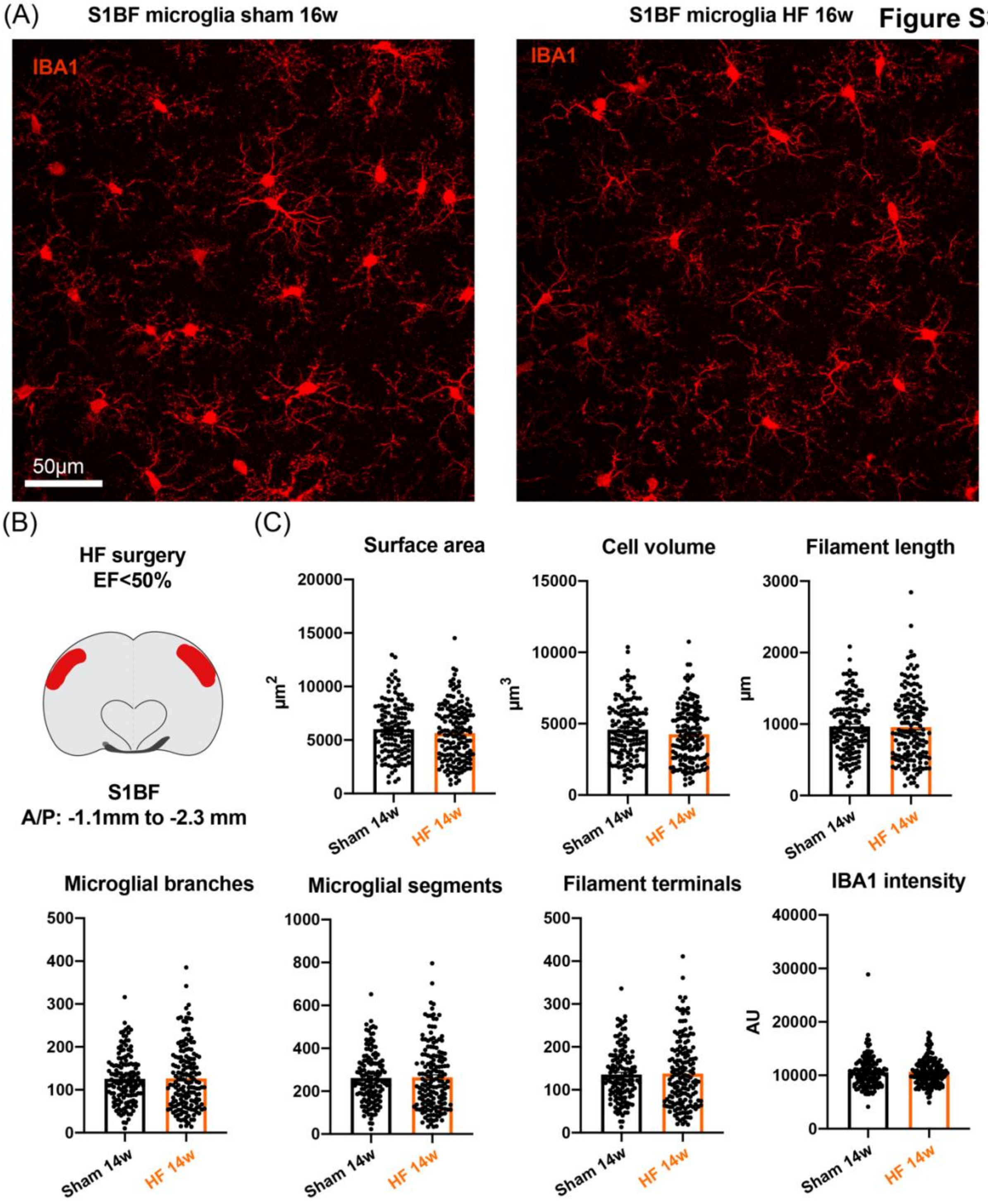
(A)
S1BF microglia sham 16w
S1BF microglia HF 16w
IBA1
IBA1
50µm
(B)
HF surgery
EF<50%
S1BF
A/P: -1.1mm to -2.3 mm
(C)
Surface area
Cell volume
Filament length
Microglial branches
Microglial segments
Filament terminals
IBA1 intensity
µm2
µm3
µm
AU
Sham 14w
HF 14w

Figure S3: HF does not induce morphometric changes in somatosensory cortex 1 barrel field microglia (a) representative confocal images show IBA1-stained microglia in the S1BF of HF and sham rats 16 weeks post-surgery, (b) brain scheme shows the topographic location of S1BF microglia that have been used for the morphometric assessment. The red area highlights the S1BF, (c) dot-plot graphs show the individual values of S1BF microglia for surface area, cell volume, filament length, microglial branches, microglial segments, filament terminals and IBA 1 intensity for sham rats (n=4) and HF rats (n= 4). One-way ANOVA was used to analyze the data.

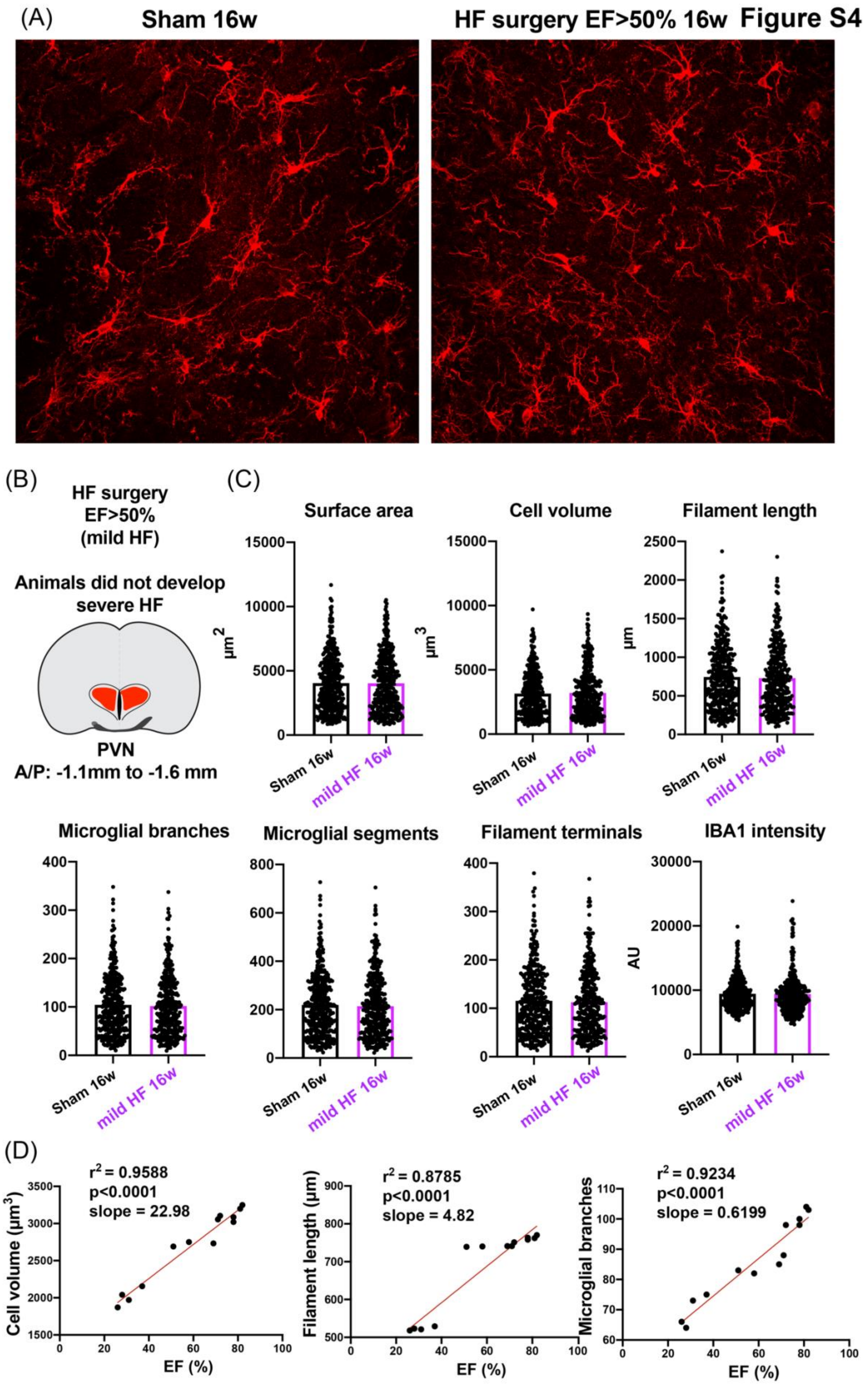

Figure S4
(A)
Sham 16w
HF surgery EF>50% 16w
(B)
HF surgery
EF>50%
(mild HF)
Animals did not develop
severe HF
PVN
A/P: -1.1mm to -1.6 mm
(C)
Surface area
Cell volume
Filament length
Microglial branches
Microglial segments
Filament terminals
IBA1 intensity
μm^2
μm^3
μm
AU
Sham 16w
mild HF 16w
(D)
r^2 = 0.9588
p<0.0001
slope = 22.98
Cell volume (μm^3)
r^2 = 0.8785
p<0.0001
slope = 4.82
Filament length (μm)
r^2 = 0.9234
p<0.0001
slope = 0.6199
Microglial branches
EF (%)

Figure S4: Morphological changes in microglia are not observed in rats with mild level of HF (a) representative confocal images show IBA1-stained microglia in the PVN of sham and rats that underwent heart failure surgery, but did not develop severe HF ('mild HF'), indicated by an ejection fraction greater than 50% (EF>50%), (b) brain scheme shows the topographic location of PVN microglia that have been used for the morphometric assessment. The red area highlights the PVN, (c) dot-plot graphs show the individual values of PVN microglia for surface area, cell volume, filament length, microglial branches, microglial segments, filament terminals and IBA 1 intensity for sham rats (n=4) and mild HF rats (n=5). One-way ANOVA was used to analyze the data, (d) plot graphs depicting cell volume, filament length and number of microglial branches as a function of %EF values combining sham (n=4), HF rats (EF<50%, n=4) and mild HF rats (EF>50%, n=5). R_2 and p values were obtained following a Pearson correlation analysis.

Figure S5

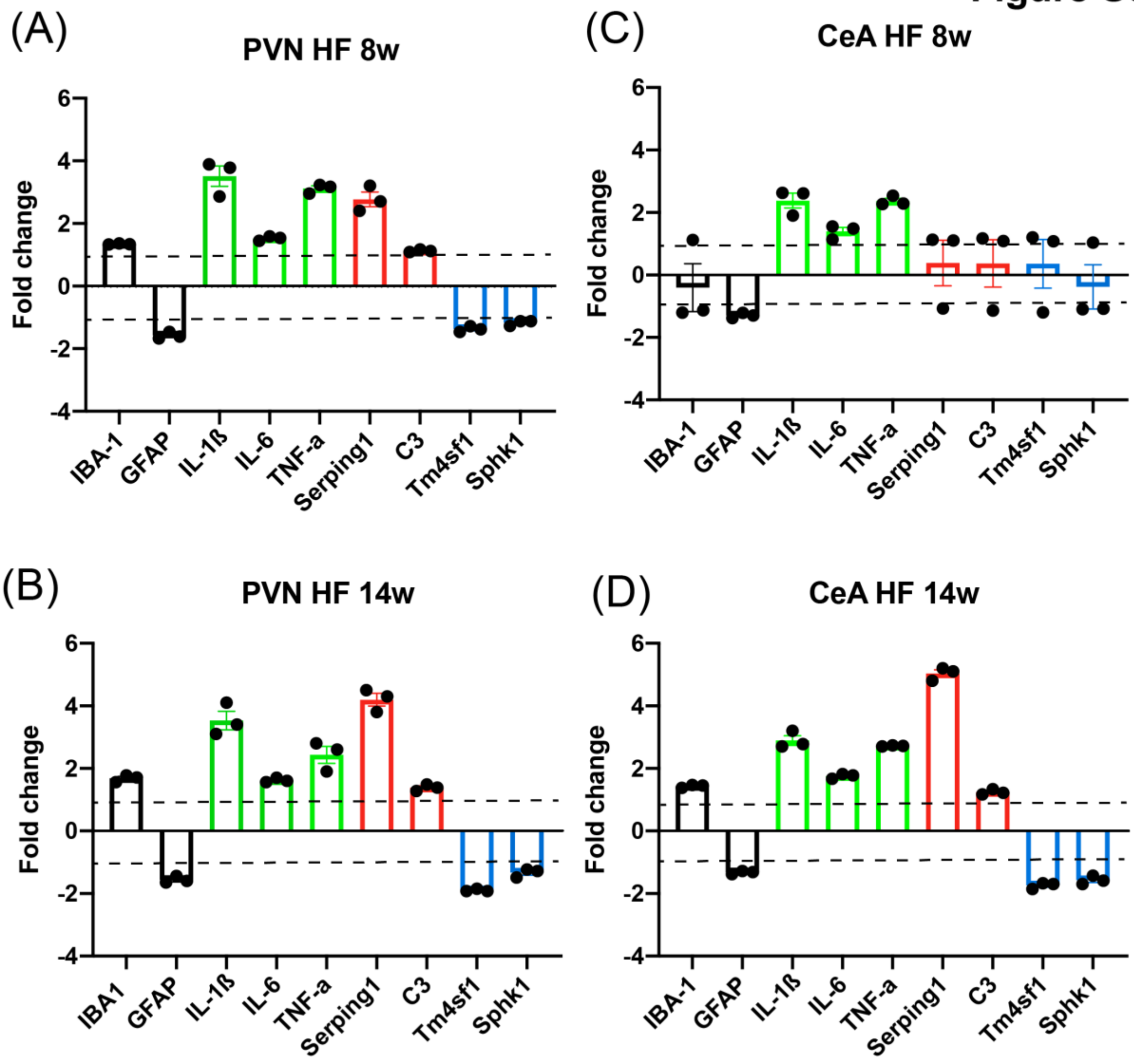

Figure S5: HF-induced changes in neuroinflammation-associated microglia and astrocyte marker mRNA levels (a-f) bar graphs show the mean fold change in mRNA transcript levels in HF rats compared to sham rats in the PVN (a, b) and the CeA (c, d). All graphs depict the fold changes of mRNA levels in HF group compared to their respective shams. Green bars: cytokines, red bars: a1 astrocyte markers, blue bars: a2 astrocyte markers. Dashed lines indicate the hypothetical mean (+1/-1) for the one-sample t-test. Each dot represents the average value of the pooled samples and the measurements were performed in triplicates. *p<0.05, **p<0.01 and ***p<0.0001 vs. respective controls, one sample t-test.
